# Mobile technologies to support healthcare provider to healthcare provider communication and management of care

**DOI:** 10.1002/14651858.CD012927.pub2

**Published:** 2020-08-18

**Authors:** Daniela C Gonçalves-Bradley, Ana Rita J Maria, Ignacio Ricci-Cabello, Gemma Villanueva, Marita S Fønhus, Claire Glenton, Simon Lewin, Nicholas Henschke, Brian S Buckley, Garrett L Mehl, Tigest Tamrat, Sasha Shepperd

**Affiliations:** Nuffield Department of Population HealthUniversity of OxfordOxfordUK; Nova Medical SchoolFaculdade de Ciências MédicasLisbonPortugal; Primary Care Research UnitInstituto de Investigación Sanitaria Illes BalearsPalma de MallorcaSpain; Cochrane ResponseCochraneLondonUK; Norwegian Institute of Public HealthOsloNorway; Department of SurgeryUniversity of the PhilippinesManilaPhilippines; Department of Sexual and Reproductive HealthWorld Health OrganizationGenevaSwitzerland; Health Systems Research UnitSouth African Medical Research CouncilCape TownSouth Africa

**Keywords:** Adult, Humans, Bias, Cell Phone, Cell Phone/statistics & numerical data, Community Health Workers, Community Health Workers/statistics & numerical data, Computer Security, Dermatologists, Diabetic Retinopathy, Diabetic Retinopathy/diagnosis, Emergency Service, Hospital, Emergency Service, Hospital/statistics & numerical data, Guideline Adherence, Guideline Adherence/statistics & numerical data, Health Care Costs, Health Personnel, Health Personnel/psychology, Health Personnel/statistics & numerical data, Health Status, Patient Satisfaction, Personal Satisfaction, Primary Health Care, Primary Health Care/statistics & numerical data, Quality of Life, Randomized Controlled Trials as Topic, Referral and Consultation, Referral and Consultation/statistics & numerical data, Renal Insufficiency, Chronic, Renal Insufficiency, Chronic/therapy, Skin Diseases, Skin Diseases/therapy, Telemedicine, Telemedicine/economics, Telemedicine/statistics & numerical data, Time Factors, Time-to-Treatment, Ultrasonography

## Abstract

**Background:**

The widespread use of mobile technologies can potentially expand the use of telemedicine approaches to facilitate communication between healthcare providers, this might increase access to specialist advice and improve patient health outcomes.

**Objectives:**

To assess the effects of mobile technologies versus usual care for supporting communication and consultations between healthcare providers on healthcare providers' performance, acceptability and satisfaction, healthcare use, patient health outcomes, acceptability and satisfaction, costs, and technical difficulties.

**Search methods:**

We searched CENTRAL, MEDLINE, Embase and three other databases from 1 January 2000 to 22 July 2019. We searched clinical trials registries, checked references of relevant systematic reviews and included studies, and contacted topic experts.

**Selection criteria:**

Randomised trials comparing mobile technologies to support healthcare provider to healthcare provider communication and consultations compared with usual care.

**Data collection and analysis:**

We followed standard methodological procedures expected by Cochrane and EPOC. We used the GRADE approach to assess the certainty of the evidence.

**Main results:**

We included 19 trials (5766 participants when reported), most were conducted in high‐income countries. The most frequently used mobile technology was a mobile phone, often accompanied by training if it was used to transfer digital images. Trials recruited participants with different conditions, and interventions varied in delivery, components, and frequency of contact. We judged most trials to have high risk of performance bias, and approximately half had a high risk of detection, attrition, and reporting biases. Two studies reported data on technical problems, reporting few difficulties.

**Mobile technologies used by primary care providers to consult with hospital specialists**

We assessed the certainty of evidence for this group of trials as moderate to low.

Mobile technologies:

‐ probably make little or no difference to primary care providers following guidelines for people with chronic kidney disease (CKD; 1 trial, 47 general practices, 3004 participants);

‐ probably reduce the time between presentation and management of individuals with skin conditions, people with symptoms requiring an ultrasound, or being referred for an appointment with a specialist after attending primary care (4 trials, 656 participants);

‐ may reduce referrals and clinic visits among people with some skin conditions, and increase the likelihood of receiving retinopathy screening among people with diabetes, or an ultrasound in those referred with symptoms (9 trials, 4810 participants when reported);

‐ probably make little or no difference to patient‐reported quality of life and health‐related quality of life (2 trials, 622 participants) or to clinician‐assessed clinical recovery (2 trials, 769 participants) among individuals with skin conditions;

‐ may make little or no difference to healthcare provider (2 trials, 378 participants) or participant acceptability and satisfaction (4 trials, 972 participants) when primary care providers consult with dermatologists;

‐ may make little or no difference for total or expected costs per participant for adults with some skin conditions or CKD (6 trials, 5423 participants).

**Mobile technologies used by emergency physicians to consult with hospital specialists about people attending the emergency department**

We assessed the certainty of evidence for this group of trials as moderate.

Mobile technologies:

‐ probably slightly reduce the consultation time between emergency physicians and hospital specialists (median difference −12 minutes, 95% CI −19 to −7; 1 trial, 345 participants);

‐ probably reduce participants’ length of stay in the emergency department by a few minutes (median difference −30 minutes, 95% CI −37 to −25; 1 trial, 345 participants).

We did not identify trials that reported on providers' adherence, participants’ health status and well‐being, healthcare provider and participant acceptability and satisfaction, or costs.

**Mobile technologies used by community health workers or home‐care workers to consult with clinic staff**

We assessed the certainty of evidence for this group of trials as moderate to low.

Mobile technologies:

‐ probably make little or no difference in the number of outpatient clinic and community nurse consultations for participants with diabetes or older individuals treated with home enteral nutrition (2 trials, 370 participants) or hospitalisation of older individuals treated with home enteral nutrition (1 trial, 188 participants);

‐ may lead to little or no difference in mortality among people living with HIV (RR 0.82, 95% CI 0.55 to 1.22) or diabetes (RR 0.94, 95% CI 0.28 to 3.12) (2 trials, 1152 participants);

‐ may make little or no difference to participants' disease activity or health‐related quality of life in participants with rheumatoid arthritis (1 trial, 85 participants);

‐ probably make little or no difference for participant acceptability and satisfaction for participants with diabetes and participants with rheumatoid arthritis (2 trials, 178 participants).

We did not identify any trials that reported on providers' adherence, time between presentation and management, healthcare provider acceptability and satisfaction, or costs.

**Authors' conclusions:**

Our confidence in the effect estimates is limited. Interventions including a mobile technology component to support healthcare provider to healthcare provider communication and management of care may reduce the time between presentation and management of the health condition when primary care providers or emergency physicians use them to consult with specialists, and may increase the likelihood of receiving a clinical examination among participants with diabetes and those who required an ultrasound. They may decrease the number of people attending primary care who are referred to secondary or tertiary care in some conditions, such as some skin conditions and CKD. There was little evidence of effects on participants' health status and well‐being, satisfaction, or costs.

## Plain language summary

**Using mobile technologies to promote communication and management of care between healthcare professionals**

**What is the aim of this review?**

We aimed to find out if healthcare workers using mHealth services through their mobile phones or other mobile devices to communicate with other healthcare workers provide quicker access to healthcare, and improve patient health outcomes. We collected and analysed all relevant research and found 19 studies.

**Key messages**

Mobile technologies probably slightly decrease the time to deliver health care, as well as the number of face‐to‐face appointments, when compared with usual care, and probably increase the number of people receiving clinical examinations for some conditions, including an eye exam for people with diabetes. Mobile technologies may have little or no impact on healthcare workers' and participants' satisfaction, health status or well‐being.

**What was studied in the review?**

Many healthcare workers work alone or have little access to colleagues and specialists. This is a common problem for healthcare workers in rural areas or low‐income countries.

One possible solution to this problem is to offer healthcare workers advice and support through mobile technologies that allow healthcare workers to get help from colleagues who are not in the same place. For instance, healthcare workers can contact specialists or colleagues with more experience through a phone or the Internet. Healthcare workers can also use their mobile phones or other mobile devices such as tablets. As more healthcare workers use mobile phones and other devices as part of their work, this could make it particularly easy for them to use mHealth services.

**What are the main results of the review?**

We found 19 relevant studies, which included more than 5766 people who needed health care. Sixteen studies were from high‐income countries. Two studies reported on technical problems, reporting few difficulties.

**When primary healthcare workers use mobile technologies to consult with hospital specialists, they:**

‐ probably make little or no difference to whether guidelines are followed for people with chronic kidney disease, or to health status or quality of life of people with psoriasis.

‐ may increase the likelihood of retinopathy screening for people with diabetes, or receiving an ultrasound if referred with symptoms, and may reduce referrals or a visit to the clinic for people with a skin condition or referred for clinic follow‐up for different health problems.

‐ may make little or no difference to healthcare worker or patient satisfaction, or to how much it costs to deliver health care.

**When emergency doctors use mobile technologies to consult with hospital specialists:**

‐ patients are probably managed slightly more quickly.

We did not find any studies that looked at the effect of mobile technologies on emergency doctors following guidelines, patients’ health and well‐being, healthcare worker or patient satisfaction, or costs.

**When community health workers or home‐care workers use mobile technologies to consult with clinic staff, they:**

‐ probably make little or no difference to the number of times people with a new diabetes‐related foot ulcer have to see a nurse, or elderly people using tube feeding have to see a nurse or go into hospital.

‐ may make no difference to the number of people living with HIV or diabetes who die; and may make little or no difference to the health status or quality of life of people with rheumatoid arthritis.

‐ probably make little or no difference to the satisfaction of people with diabetes or rheumatoid arthritis.

We did not find any studies that looked at the effect of mobile technologies on whether community health workers follow guidelines, how quickly people receive care, healthcare worker satisfaction, costs, or technical difficulties.

**How up‐to‐date is this review?**

We searched for studies up to 22 July 2019.

## Summary of findings

**Summary of findings 1:** Mobile technologies used by primary care providers to consult with a hospital‐based specialist compared with usual care

**Mobile technologies used by primary care providers to consult with a hospital‐based specialist compared with usual care**				
**Population:** Primary care providers consulting with dermatologists (6 studies), ophthalmologists (2 studies), radiologists (1 study), nephrologists (1 study), or different specialists (1 study) **Setting:** Primary care settings in North America (5 studies), Europe (4 studies), the Dominican Republic (1 study) or Mongolia (1 study) **Intervention:** Mobile technologies for retinal screening using a non‐mydriatic camera (2 studies), portable ultrasound (1 study), teledermatology to send digital images (6 studies), eConsult through audio‐conferencing or secure direct messaging between healthcare providers (2 studies) **Comparison:** Usual care that included a reminder to book an appointment with participant’s healthcare provider; direct booking of a face‐to‐face appointment; regular examination during the index face‐to‐face appointment with the participant’s primary care provider				
**Outcomes**	**Impact**	**№ of participants (studies)**	**Certainty of the evidence (GRADE)**	**Plain language statement**
Providers' adherence to recommended practice, guidelines or protocols: Adherence to the advised monitoring criteria Follow‐up not specified	1 trial of telenephrology (Van Gelder 2017), using a web‐based platform with access to the electronic medical record reported OR of 1.23 (95% CI 0.89 to 1.70) for monitoring of disease and 0.61 (0.22 to 1.72) for monitoring of metabolic parameters	3004 (1 cluster‐randomised trial, 47 general practices)	⊕⊕⊕⊝ Moderate^a^	Mobile technologies used by primary care providers to consult with a hospital‐based specialist probably make little or no difference to primary care providers’ adherence to the advised monitoring criteria for participants with chronic kidney disease (CKD), when compared with usual care
Time between presentation and management of the health condition Follow‐up: 3 to 6 months	2 trials of teledermatology (Piette 2017; Whited 2002) reported that participants allocated to IG received the required treatment in less time than those allocated to CG (median delay 4 days for IG and 40 days for CG; MD −40.5 days, 95% CI −23 to −58) 1 trial of telemedicine using a portable ultrasound (Sutherland 2009) for people presenting with symptoms that required an ultrasound reported little or no difference between groups. 1 trial of eConsult for people attending primary care (Azogil‐López 2019) reported that participants allocated to IG had an appointment in less time than those allocated to CG (median difference −27 days, 99% CI −20 to −33)	656 (4 randomised trials)	⊕⊕⊕⊝ Moderate^b^	The intervention probably reduces time between participants presenting and management among individuals with some skin conditions, symptoms requiring an ultrasound, or requiring an appointment with a specialist after attending primary care
Healthcare use Follow‐up: 3 to 12 months	4 trials of teledermatology (Byamba 2015; Piette 2017; Whited 2002; Whited 2013; RRs ranged from to 0.28 (95% CI 0.13 to 0.63) to 0.82 (95% CI 0.75 to 0.88)) reported that those participants allocated to the intervention group were less likely to be referred for clinic follow‐up or attend an appointment at a clinic 2 trials of eConsults for nephrology (Van Gelder 2017) and different specialties (Liddy 2019a) reported little or no difference between groups (OR 0.61, 95% CI 0.31 to 1.23 and RR 0.93, 95% CI 0.85 to 1.03, respectively) 2 trials of telemedicine for retinopathy screening (Davis 2003; Mansberger 2015) and 1 trial for people presenting with symptoms that required an ultrasound (Sutherland 2009; RR 3.92, 95% CI 2.11 to 7.31) reported that those participants allocated to the intervention group were more likely to receive a clinical examination	4810 (9 randomised trials)	⊕⊕⊕⊝ Moderate^c^	Mobile technologies used by primary care providers to consult with hospital‐based specialists may reduce referrals and clinic visits among people with skin conditions, and increase the likelihood of receiving retinopathy screening among participants with diabetes, and an ultrasound in those referred with symptoms, when compared with usual care 1 trial did not specifically report the number of participants involved
Participants' health status and well‐being	Patient‐reported quality of life and health‐related quality of life (Follow‐up: 9 to 12 months)			
	2 trials of teledermatology (Armstrong 2018; Whited 2013) found little or no difference between groups For health status (EQ‐5D‐5L): MD 0 (95% CI −0.003 to 0.003) For quality of life (Skindex‐16): IG: MD −12.0 (SD 24.5, 160 participants), CG: MD −13.2 (SD 21.6, 164 participants) For health‐related quality of life (SF‐12), results reported as little or no difference between groups	622 (2 randomised trials)	⊕⊕⊕⊝ Moderate^d^	Mobile technologies used by primary care providers to consult with hospital‐based specialists probably make little or no difference to quality of life and health‐related quality of life among individuals with skin conditions
	Clinician‐assessed clinical course (follow‐up: 4 to 9 months)			
	2 trials of teledermatology (Pak 2007; Whited 2013) found little or no difference between groups	769 (2 randomised trials)	⊕⊕⊕⊝ Moderate^e^	Mobile technologies used by primary care providers to consult with hospital‐based dermatologists probably make little or no difference to clinical improvement among individuals with skin conditions
Acceptability and satisfaction	Healthcare provider acceptability and satisfaction (follow‐up immediately after the intervention)			
	1 trial of teledermatology (Piette 2017) reported little or no difference between groups 1 trial of teledermatology (Whited 2002) reported that GPs allocated to the intervention were more likely to agree that participants received timely appointments and to be satisfied with the consult process than GPs allocated to the control group	378 (2 randomised trials)	⊕⊕⊝⊝ Low^f^	Mobile technologies used by primary care providers to consult with hospital‐based dermatologists may make little or no difference to healthcare provider acceptability and satisfaction with the intervention
	Participant acceptability and satisfaction (follow‐up: 1 to 9 months)			
	4 trials of teledermatology (Eminović 2009; Piette 2017; Whited 2002; Whited 2013) reported little or no difference between groups 1 trial reported MD 0.0 (95% CI −0.12 to 0.12; PSQ III), another trial reported that 87% of participants allocated to the intervention group were overall satisfied with treatment received, compared with 92% of those allocated to the control group* 2 trials reported the results as little or no difference only (VSQ9; *)	972 (4 randomised trials)	⊕⊕⊝⊝ Low^g^	Mobile technologies used by primary care providers to consult with hospital‐based dermatologists may make little or no difference to acceptability and satisfaction of participants with skin conditions
Costs Follow‐up: 1 to 9 months	2 teledermatology trials (Eminović 2009; Whited 2013) and 1 telenephrology trial (Van Gelder 2017) reported little or no difference between groups 2 teledermatology trials (Pak 2007; Whited 2002) reported that when loss of productivity was considered, the cost per participant was higher for those allocated to the intervention 1 trial of teledermatology (Byamba 2015) reported that total costs were lower for those allocated to the intervention group.	5423 (6 randomised trials)	⊕⊕⊝⊝ Low^h^	The intervention may make little or no difference to total or expected costs per participant for adults with skin conditions or chronic kidney disease
Technical problems	1 trial recruiting GPs consulting with dermatologists about images they took (Pak 2007) reported that there was little or no difference between groups for technical problems	698 (1 randomised trial)	⊕⊕⊕⊝ Moderate^i^	The intervention probably results in few or no technical difficulties
**CG:** Control group; **CI:** Confidence interval; **EQ5D:** EuroQol five dimensions questionnaire; **GPs:** General practitioners; **IG:** Intervention group; **MD:** Median difference; **OR:** Odds ratio; **PSQ III:** Shortened version of the Patient Satisfaction Questionnaire; **RR:** Risk ratio; **SD:** Standard deviation; **SF‐12:** Short‐Form Health Survey 12; **VSQ9:** Visit‐specific satisfaction questionnaire (VSQ9) * Questions developed by the authors for the specific trial				
**GRADE Working Group grades of evidence** **High certainty:** We are very confident that the true effect lies close to that of the estimate of the effect **Moderate certainty:** We are moderately confident in the effect estimate: The true effect is likely to be close to the estimate of the effect, but there is a possibility that it is substantially different **Low certainty:** Our confidence in the effect estimate is limited: The true effect may be substantially different from the estimate of the effect **Very low certainty:** We have very little confidence in the effect estimate: The true effect is likely to be substantially different from the estimate of effect				

**Rationale for downgrading the evidence** ^a^We downgraded one point for risk of bias due to performance and detection bias, and lack of protection against contamination. ^b^We downgraded one point for risk of bias due to high risk of selection bias (2 trials), performance bias (3 trials), and reporting (2 trials) bias. ^c^We downgraded one point for risk of bias due to high risk of selection (2 trials), performance (6 trials), detection (3 trials), attrition (1 trial) and reporting (2 trial) bias. ^d^We downgraded one point for risk of bias due to high risk of performance (2 trials), detection (2 trials), and reporting (2 trials) bias. ^e^We downgraded one point for risk of bias due to high risk of performance, attrition and reporting bias. ^f^We downgraded two points for risk of bias due to high risk of selection (1 trial), performance (2 trials), detection (2 trials), and reporting (1 trial) bias. ^g^We downgraded two points for risk of bias due to high risk of selection (1 trial), performance (4 trials), detection (4 trials), attrition (1 trial) and reporting (3 trials) bias. ^h^We downgraded two points for risk of bias due to high risk of detection (2 trials), performance (6 trials), selection (1 trial), attrition (2 trials), contamination (1 trial) and reporting bias (4 trials). ^i^We downgraded one point for risk of bias due to high risk of performance, reporting and attrition bias.

**Summary of findings 2:** Mobile technologies for use in the emergency department compared with usual care

**Mobile technologies for use in the emergency department compared with usual care**					
**Patient or population:** Emergency physicians consulting with hospital specialists about adults attending the emergency department **Setting:** Turkey **Intervention:** Smartphone application for secure messaging, including clinical images **Comparison:** Usual care ‐ consultation requests were done by telephone, with any clinical information sent verbally					
**Outcomes**	**Impact**	**№ of participants (studies)**	**Certainty of the evidence (GRADE)**	**Plain language statement**	
Providers' adherence to recommended practice, guidelines or protocols	‐	‐	‐	No studies were identified	
Time between presentation and management of the health condition Follow‐up not reported	1 trial (Gulacti 2017) reported that those allocated with the intervention group were admitted to hospital or discharged more quickly from the emergency department (median difference −12 minutes, 95% CI −19 to −7 minutes)	345 (1randomised trial)	⊕⊕⊕⊝ Moderate^a^	The intervention probably reduces time between participants presenting and management by a few minutes among individuals visiting the emergency department	
Healthcare use: length of stay in the emergency department Follow‐up not reported	1 trial (Gulacti 2017) reported that participant allocated to the intervention group participants had a shorter stay in the emergency department (median difference −30 minutes, 95% CI: −37 to −25 minutes)	345 (1 randomised trial)	⊕⊕⊕⊝ Moderate^a^	The intervention probably slightly reduces length of stay among individuals visiting the emergency department	
Participants' health status and well‐being	‐	‐	‐	No studies were identified	
Participant and provider acceptability or satisfaction	‐	‐	‐	No studies were identified	
Costs	‐	‐	‐	No studies were identified	
Technical problems	1 trial (Gulacti 2017) reported that there were no technical problems during the course of the trial	345 (1 randomised trial)	⊕⊕⊕⊝ Moderate^a^	The intervention probably results in few or no technical difficulties	
**CI:** Confidence interval					
**GRADE Working Group grades of evidence** **High certainty:** We are very confident that the true effect lies close to that of the estimate of the effect **Moderate certainty:** We are moderately confident in the effect estimate: The true effect is likely to be close to the estimate of the effect, but there is a possibility that it is substantially different **Low certainty:** Our confidence in the effect estimate is limited: The true effect may be substantially different from the estimate of the effect **Very low certainty:** We have very little confidence in the effect estimate: The true effect is likely to be substantially different from the estimate of effect					

**Rationale for downgrading the evidence** ^a^We downgraded one point for risk of bias due to high risk of performance and reporting bias.

**Summary of findings 3:** Mobile technologies used by community health or home‐care workers compared with usual care

**Mobile technologies used by community health or home‐care workers compared with usual care**				
**Patient or population:** Community‐based peer health workers consulting with clinic staff about receiving antiretroviral therapy, community nurses consulting with diabetes specialist nurses or podiatrists about adults with Type 2 diabetes, home‐care nurses consulting with hospital specialists about home enteral nutrition, rural‐based physical therapists consulting with urban‐based rheumatologists **Setting:** Canada, Italy, Norway, Uganda **Intervention:** Mobile technologies (teledermatology, mobile text messaging, interactive web‐based records, video‐consultations) **Comparison:** Usual care ‐ home visits or outpatient clinics				
**Outcomes**	**Impact**	**№ of participants (studies)**	**Certainty of the evidence (GRADE)**	**Plain language statement**
Providers' adherence to recommended practice, guidelines or protocols	‐	‐	‐	No studies were identified
Time between presentation and management of the health condition	‐	‐	‐	No studies were identified
Healthcare use	Outpatient clinic and community nurse consultations (follow‐up: 12 months)			
	2 trials (Iversen 2018; Orlandoni 2016) reported little or no difference between groups for outpatient visits (MD −0.48, 95% CI −1.46 to 0.49) or community nurse consultations (MD 0.92, 95% CI −0.70 to 2.53)	370 (2 randomised trials)	⊕⊕⊕⊝ Moderate^a^	Mobile technologies used by community health or home‐care workers probably make little or no difference for outpatient clinic and community nurse consultations of participants with new diabetes‐related foot ulcer and older individuals treated with home enteral nutrition
	Hospitalisation (Follow‐up: 12 months)			
	1 study (Orlandoni 2016) reported that the incidence rate ratio for hospitalisations was similar between groups among older individuals treated with home enteral nutrition (95% CI 0.54 to 1.19, P = 0.26)	188 (1 randomised trial)	⊕⊕⊝⊝ Low^b, c^	Mobile technologies for communication between home‐visiting nursing staff consulting with a hospital physician may have little or no effect on hospitalisations among older individuals treated with home enteral nutrition
Participants' health status and well‐being	Mortality among individuals living with HIV or diabetes (Follow‐up: 11 to 12 months)			
	2 trials reported little or no differences between groups. 1 study (Chang 2011) recruited peer health workers who consulted with clinic staff (RR: 0.82, 95% CI 0.55 to 1.22), and another study (Iversen 2018) recruited community nurses who consulted with diabetes specialist nurses (RR: 0.94, 95% CI 0.28 to 3.12).	1157 (2 randomised trials)	⊕⊕⊝⊝ Low^d, e^	The intervention may make little or no difference in mortality among people living with HIV or diabetes
	Disease activity or health‐related quality of life (Follow‐up: 9 months)			
	1 trial of rural‐based physical therapists consulting with urban‐based rheumatologists about adults with a clinical diagnosis of rheumatoid arthritis (Taylor‐Gjevre 2018) reported little or no difference between groups for disease activity (DAS28‐CRP MD 0.9, 95% CI −1.2 to 3.1; mHAQ MD 0.2, 95% CI −0.1 to 0.5; RADAI MD 0.9, 95% CI −0.5 to 2.4) or health‐related quality of life (EQ5D MD −0.1, 95% CI −0.4 to 0.1)	85 (1 randomised trial)	⊕⊕⊝⊝ Low^b^,^f^	Mobile technologies used by community health or home‐care workers may make little or no difference for disease activity and health‐related quality of life in participants with rheumatoid arthritis
Participant and provider acceptability or satisfaction	Healthcare provider acceptability and satisfaction			
	‐	‐	‐	No studies were identified
	Participant acceptability and satisfaction (Follow‐up: 9 to 12 months)			
	2 trials on diabetes (Iversen 2018) and arthritis (Taylor‐Gjevre 2018) reported little or no difference between groups for participants' experience with healthcare (GS‐PEQ MD 0.0, 95% CI −0.18 to 0.18) and satisfaction (VSQ9 results reported narratively) with the intervention.	178 (2 randomised trials)	⊕⊕⊕⊝ Moderate^g^	Mobile technologies used by community health or home‐care workers probably make little or no difference for participant acceptability and satisfaction for participants with new diabetes‐related foot ulcer and participants with rheumatoid arthritis
Costs	‐	‐	‐	No studies were identified
Technical difficulties	‐	‐	‐	No studies were identified
**CI:** Confidence interval; **DAS28‐CRP:** Disease activity score for Rheumatoid Arthritis; **EQ5D:** EuroQol five dimensions questionnaire; **GS‐PEQ:** Generic Short Patient Experiences Questionnaire; **MD:** Mean difference; **mHAQ:** Modified health assessment questionnaire; **RADAI:** Rheumatoid arthritis disease activity index; **RR:** Risk ratio; **VSQ9:** Visit‐specific satisfaction questionnaire				
**GRADE Working Group grades of evidence** **High certainty:** We are very confident that the true effect lies close to that of the estimate of the effect **Moderate certainty:** We are moderately confident in the effect estimate: The true effect is likely to be close to the estimate of the effect, but there is a possibility that it is substantially different **Low certainty:** Our confidence in the effect estimate is limited: The true effect may be substantially different from the estimate of the effect **Very low certainty:** We have very little confidence in the effect estimate: The true effect is likely to be substantially different from the estimate of effect				

**Rationale for downgrading the evidence** ^a^We downgraded one point for risk of bias due to high risk of performance (2 studies), detection (2 studies), attrition (1 study) and reporting (1 study) bias. ^b^We downgraded one point for imprecision because the 95% CI shows potential effect on both sides of “no effect” line and that there were few events. ^c^We downgraded one point for risk of bias due to high risk of performance, detection, and attrition bias. ^d^We downgraded one point for imprecision because the 95% CI shows potential effect on both sides of “no effect” line . ^e^We downgraded one point for risk of bias due to high risk of performance (2 studies), detection (1 study), attrition (1 study) and reporting (2 studies) bias. ^f^We downgraded one point for risk of bias due to high risk of performance, detection, attrition, and reporting bias. ^g^We downgraded one point for risk of bias due to high risk of performance (2 studies), detection (2 studies), attrition (1 study), and reporting (2 studies) bias.

## Background

Effective communication with other healthcare providers and access to specialist expertise is essential for increasing health services capacity and providing optimal care, especially in areas where there is a shortage of healthcare providers (AAP 2015). The widespread use of information and communication technologies (ICT) can potentially increase the capacity of health services by supporting communication between different providers, and providing rapid access to specialist expertise.

### Description of the condition

By 2035 there will be a worldwide shortage of approximately 12.9 million skilled healthcare providers (Campbell 2013). The biggest gaps will occur in Southeast Asia and sub‐Saharan Africa, but elsewhere too this will be a problem due to larger ageing populations, the rising prevalence of non‐communicable diseases, migration patterns and high turnover of healthcare providers. Remote and rural areas, where populations are likely to be poorer, sicker and less educated, are particularly at risk (OPHI 2017; Wu 2016). Healthcare providers in those settings can be isolated and have limited interaction with colleagues and specialists, with few opportunities for mentoring, consultation with experts, or referrals to other healthcare providers.

### Description of the intervention

Digital technologies are increasingly used to support health systems (WHO 2018) by providing flexible options for communication and the exchange of information. These technologies can be used for medical diagnostic, monitoring and therapeutic purposes, when participants are separated by distance or time or both, with the ultimate goal of improving the health of individuals and communities (Steinhubi 2013). Provision of health care at a distance is usually referred to as telemedicine (WHO 2018), and can be implemented through mobile or fixed devices.

The exchange of information can happen synchronously (when interactions happen in real time) or asynchronously (when there is a lag between the clinical information being transmitted and the response), and through different channels, including video‐conferencing, mobile applications, and secure messaging (Kruse 2017; WHO 2016). The use of mobile technologies can improve access to specialty care (Liddy 2019b), particularly for underserved communities (Källander 2013). Widespread mobile broadband connectivity means that even healthcare providers in remote areas can access and communicate with their peers, improving co‐operation (Aceto 2018). The World Health Organization (WHO) Global Observatory for eHealth conducted a survey of the WHO Member States on the use of eHealth (WHO 2016), and reported that of the 122 countries surveyed 70% reported on the use of mobile health devices for consultation between healthcare professionals. The most common areas were teleradiology, telepathology, and teledermatology (WHO 2016), with teleradiology programmes being widely used. Within this review our focus was on mobile technologies to support provider‐to‐provider communication and management of care.

In a bid to maximise the coverage of healthcare services and to decrease the cost of providing health care, governments and healthcare agencies in some countries have funded some type of telehealth programme for provision of care, including promoting communication and management of care between providers. Some examples include the Technology Enabled Care Services programme in England (NHS Commissioning Assembly 2015), the Scottish Centre for Telehealth and Telecare (SCTT 2017), the telehealth services provided within the Medicare programme in the USA (MedPAC 2016), the Asia eHealth Information Network (AeHIN 20017), the KwaZulu‐Natal Experience in South Africa (Mars 2012), and the Aga Khan Development Network Digital Health Programme, which covers remote communities in South‐Central Asia and East Africa (AKDN 2019).

### How the intervention might work

The use of mobile technologies between healthcare providers for communication, consultations and patient management might contribute to developing professional skills and expertise, as well as optimising multidisciplinary communication (AAP 2015) and evidence‐based clinical practice. This is particularly relevant for settings where there is a shortage of healthcare providers, for instance in low‐ and middle‐income countries and in rural and remote areas (Källander 2013). By enabling healthcare providers who are geographically separated to exchange clinical information and knowledge, mobile technology can facilitate universal health coverage by increasing access to health care. In 2018 the WHO published a classification of digital health interventions to categorise the functionality of the different applications; using this classification as a guide we include interventions that are portable and facilitate remote healthcare provider communication or co‐ordination of referrals, or both (WHO 2018).

Despite the possibilities, telehealth applications have been inconsistently implemented, with varying degrees of success due to technological challenges, legal considerations, human and cultural factors, and uncertainty around economic benefits and cost effectiveness (WHO 2016), although this is changing. Overcoming these barriers requires evidence‐based implementation of guidelines, driven both by governmental and professional medical organisations; legislation on confidentiality, privacy and liability; and the involvement of stakeholders in designing, implementing and evaluating telemedicine applications, focusing on the safety and the effectiveness of applications (Agboola 2016).

### Why it is important to do this review

The rapid progress of information and communication technologies is accelerating the evolution of remote communication between providers for the management of care. This review is one of a suite of 11 Cochrane Reviews that contributed to the WHO guideline on digital interventions for health systems strengthening (WHO 2019), and focuses on the effectiveness of mobile technologies for communication and management of care between healthcare providers who are in different locations. The effectiveness of mobile technologies to support patient‐to‐healthcare provider communication is being assessed in another review (Gonçalves‐Bradley 2018a). The rationale for conducting this review is to assess the effectiveness of mobile health technologies as a method for healthcare providers to communicate, diagnose and manage patients; and to assess acceptability, satisfaction, resource use and technical difficulties. Research into the latter has been particularly neglected (Coiera 2016), and can provide crucial information for successful implementation.

## Objectives

To assess the effects of mobile technologies versus usual care for supporting communication and consultations between healthcare providers on healthcare providers' performance, acceptability and satisfaction, healthcare use, patient health outcomes, acceptability and satisfaction, costs, and technical difficulties.

## Methods

### Criteria for considering studies for this review

#### Types of studies

We include randomised trials reported as full‐text studies, conference abstracts and unpublished data, irrespective of their publication status and language of publication.

#### Types of participants

All types of healthcare providers (i.e. professionals, healthcare assistants, and lay health workers) providing patient care through mobile technologies. We included trials targeting people with any condition, regardless of their location, setting, diagnoses, or demographic factors such as age.

#### Types of interventions

We include trials comparing health care delivered through a mobile device versus usual care. We defined 'usual care' by the setting in which the trial took place, including face‐to‐face exchanges and communication through other non‐digital channels. We include trials of healthcare providers who were geographically separated and used information and communication technologies. We have focused exclusively on the exchange of clinical information over wireless and mobile technologies, mobile phones of any kind (but not analogue land‐line telephones), tablets, personal digital assistants and smartphones, and when the healthcare provider enquiry received a response in real‐time or as immediate as clinically appropriate. Communication channels through a mobile device can include text messaging, video messaging, social media, voice calls, voice‐over Internet protocol (VoIP), and video‐conferencing, through software such as Skype, WhatsApp or Google Hangouts.

We include:

- trials in which the healthcare provider used mobile technologies, such as telemedicine applications, to seek clinical guidance and support from other qualified healthcare providers in order to deliver direct patient care. This included co‐ordination of referrals and requests for expert opinion and diagnosis;
- trials in which the provider(s) seeking guidance was at a different location from the provider(s) offering guidance; and
- trials in which the provider(s) seeking guidance transmitted clinical information using a mobile device and the provider(s) offering guidance responded on any device, including stationary devices.

We include trials of telemedicine interventions if they were portable/mobile. We include trials assessing unspecified types of communication devices for transmitting clinical information, so long as they were mobile, since trials often failed to report this detail.

We include all health issues and did not restrict the content of clinical health information exchanged. We include trials where the digital component of the intervention was delivered as part of a wider package if we judged it to be the core component of the intervention.

We excluded:

- pilot and feasibility studies (pilot study defined as "a version of the main study that is run in miniature to test whether the components of the main study can all work together" and feasibility studies as "pieces of research done before a main study"; Arain 2010);
- trials that compared different technical specifications of telecommunication technologies (e.g. different communication channels, software, etc.);
- trials in which the use of telecommunications technology was not directly linked to patient care;
- trials in which the primary purpose of the intervention was education/training;
- trials assessing the accuracy of a portable medical device.

#### Types of outcome measures

##### Main outcomes

- Providers' adherence to recommended practice, guidelines or protocols.
- Time between presentation and management of the health condition.

##### Other outcomes

- Healthcare use, including referrals, clinical examinations and hospitalisations.
- Participants' health status and well‐being, to include mortality and measures of health status such as the Nottingham Health Profile or the SF‐36 (McDowell 2006).
- Healthcare provider acceptability and satisfaction; this includes self‐reported acceptability and satisfaction, measured with a validated scale, such as the Physician Worklife Survey (Konrad 1999).
- Participant acceptability and satisfaction; this included self‐reported acceptability and satisfaction, measured with a validated scale, such as the Patient Satisfaction Scale (La Monica 1986).
- Costs, including cost to the user and cost to the service (e.g. human resources/time, training, supplies and equipment).
- Unintended consequences; these could include errors in interpreting the data; transmission of inaccurate data, loss of verbal and non‐verbal communication cues, issues of privacy and disclosure that might affect interpersonal relationships, negative impacts on equity, and technical difficulties, for example failure or delay in the message delivery.

### Search methods for identification of studies

#### Electronic searches

An Information Specialist developed the search strategies in consultation with the review authors and WHO content experts. We used a minimum cut‐off search date of 2000, based on the increased availability and penetration of mobile devices from that date onwards (ITU 2019). Appendix 1 lists the search strategies and results.  We searched the following databases until 22 July 2019:

- Cochrane Central Register of Controlled Trials (CENTRAL; 2019, Issue 7), in the Cochrane Library;
- MEDLINE Ovid;
- Embase Ovid;
- POPLINE;
- WHO Global Health Library.

#### Searching other resources

##### Trial registries

We searched clinicaltrials.gov (clinicaltrials.gov) and the World Health Organization International Clinical Trials Registry Platform (who.int/ictrp).

##### Grey literature

We conducted a grey literature search in August 2017, to identify trials not indexed in the databases listed above. We searched for relevant systematic reviews and primary studies on similar topics using Epistemonikos (epistemonikos.org), a database of health evidence and health‐related systematic reviews. We searched the content in mHealthEvidence (mhealthevidence.org), a database of global literature on mHealth. We contacted authors of relevant trials/reviews to clarify reported published information and to seek unpublished results/data, as well as researchers with expertise relevant to the review topic. Moreover, WHO issued a call for papers through popular digital health communities of practice such as the Global Digital Health Network and Implementing Best Practices, to identify additional primary trials as well as grey literature. We performed a backward and forward search of the primary reference identified for each eligible trial.

### Data collection and analysis

#### Selection of studies

We downloaded all titles and abstracts retrieved by electronic searching to reference management databases (Distiller and Covidence) and removed duplicates. For title and abstract screening, we used a machine‐learning classifier that is able to assign a probability score that a given record describes or does not describe a randomised trial (Wallace 2017). Two review authors (from AM, BB, DGB, GV, IRC, and NH) screened titles and abstracts of trials with at least a 10% probability of being a randomised trial, and one review author screened those with less than a 10% probability. We retrieved the full‐text trial reports/publication of all potentially eligible reports, and two review authors (from AM, BB, DGB, GV, IRC, and NH) screened the full text to identify trials for inclusion and to identify and record reasons for excluding the ineligible trials. We resolved any disagreement through discussion, and if required consulted a third review author (DGB or SS).

We listed trials that initially appeared to meet the inclusion criteria but that we later excluded in the Characteristics of excluded studies table. We collated multiple reports of the same trial so that each trial rather than each report was the unit of interest in the review. We also provided any information we could obtain about ongoing studies. We recorded the selection process in sufficient detail to complete a PRISMA flow diagram (Liberati 2009).

#### Data extraction and management

We used the EPOC standard data collection form and adapted it for trial characteristics and outcome data (EPOC 2017a); we piloted the form on five trials. One review author extracted the following characteristics and a second review author cross‐checked data (from AM, BB, DGB, GV, IRC, and NH).

- Methods: trial design, unit of allocation, location and trial setting, withdrawals.
- Participants: number, mean age, age range, sex, inclusion criteria, exclusion criteria, dates conducted, other relevant characteristics.
- Interventions: function of the intervention (monitoring, consultation, therapy), intervention components (including type of technology and mode of delivery, frequency of data transmission), comparison, fidelity assessment. For this review, we defined monitoring as the continuous evaluation of the progress of symptoms or a condition over a period of time; consultation as an exchange between the healthcare provider and the participant, where the provider discusses the participant's health status and provides guidance, support, or information; and therapy as the ongoing management and care of a participant, to counteract a disease or disorder.
- Outcomes: main outcomes specified and collected, time points reported.
- Notes: funding for trial, ethical approval.

We contacted authors of included trials to seek missing data. We noted in the Characteristics of included studies table if outcome data were reported in an unusable way. We resolved disagreements by consensus or by involving a third review author (DGB or SS). We used Review Manager 5 (RevMan 5.3) for data management.

#### Assessment of risk of bias in included studies

One review author assessed risks of bias for each trial using the criteria outlined in the *Cochrane Handbook for Systematic Reviews of Interventions* (Higgins 2017), plus the guidance from the EPOC group (EPOC 2017b), and a second review author cross‐checked data (from AM, BB, DGB, GV, IRC, and NH). We resolved any disagreement by discussion or by involving a third review author (DGB or SS). We assessed the risks of bias according to the following domains.

- Random sequence generation.
- Allocation concealment.
- Blinding of participants and personnel.
- Blinding of outcome assessment.
- Incomplete outcome data.
- Selective outcome reporting.
- Baseline outcomes measurement.
- Baseline characteristics.
- Other bias.

We judged the risk of each potential source of bias as being high, low or unclear, and provide a quotation from the trial report together with a justification for our judgement in the 'Risk of bias' table. We summarised the 'Risk of bias' judgements across different trials for each of the domains listed. We considered blinding separately for different key outcomes where necessary (e.g. for unblinded outcome assessment, risk of bias for all‐cause mortality may be very different than for a participant‐reported pain scale). We assessed incomplete outcome data separately for different outcomes. Where information on risk of bias relates to unpublished data or correspondence with a trialist, we noted this in the 'Risk of bias' table. We did not exclude trials on the grounds of their risk of bias but clearly reported the risk of bias when presenting the results of the trials.

When considering treatment effects, we took into account the risk of bias for the trials that contributed to that outcome.

We conducted the review according to the published protocol (Gonçalves‐Bradley 2018b) and reported any deviations from it in 'Differences between protocol and review'.

#### Measures of treatment effect

We estimated the effect of the intervention using risk ratios (RRs) and associated 95% confidence intervals (CIs) for dichotomous data. For continuous measures, we analysed the data based on the mean, standard deviation (SD) and number of people assessed to calculate the mean difference (MD) and 95% CI (Higgins 2019). We ensured that readers could interpret an increase in scores for continuous outcomes in the same way for each outcome, explained the direction of effect, and reported where the direction was reversed if this was necessary.

#### Unit of analysis issues

Six trials used a cluster design (Byamba 2015; Chang 2011; Eminović 2009; Iversen 2018; Piette 2017; Van Gelder 2017). Of those trials, all except one had controlled for unit‐of‐analysis errors by adjusting for clustering, and thus were not further re‐analysed.

We had planned to control for unit of analysis errors by re‐analysing the data after adjusting for clustering, using the intracluster correlation coefficient reported by the trials. When not reported, we calculated intracluster correlation coefficients estimates (Campbell 2000) and the formula 1+(M‐1)xICC, where M is the average cluster size (Higgins 2019). However, it was not possible to obtain average cluster size for Byamba 2015 and as such it is possible that there are potential unit of analysis errors associated with the effect estimates of that trial.

#### Dealing with missing data

We contacted investigators in order to verify key trial characteristics and obtain missing outcome data where possible (e.g. when a trial report was only available as an abstract). Whenever it was not possible to obtain data, we reported the level of missingness and considered how that might have impacted the certainty of the evidence.

#### Assessment of heterogeneity

We conducted meta‐analyses and calculated the I^2^ statistic to measure heterogeneity among the trials in each analysis. We considered an I^2^ value of 50% or more to represent substantial levels of heterogeneity, but this value was interpreted in light of the size and direction of effects and the strength of the evidence for heterogeneity, based on the P value from the Chi^2^ test (Deeks 2017). We identified substantial heterogeneity for one of the outcomes (mortality), but were not able to explore it by prespecified subgroup analysis as there were not enough trials.

#### Assessment of reporting biases

We attempted to contact trial authors, asking them to provide missing outcome data. Where this was not possible, and we considered that the missing data might have introduced serious bias, we explored the impact of including such trials in the overall assessment of results**.** We were not able to explore possible publication bias through a funnel plot (Sterne 2011), as we did not combine a sufficient number of trials.

#### Data synthesis

We undertook meta‐analyses for outcomes when the interventions, participants, and underlying clinical question were similar enough for pooling to make sense (Borenstein 2009). As there was considerable heterogeneity, we applied a random‐effect model (Deeks 2017). A common way that trialists indicate the presence of skewed data is by reporting medians and interquartile ranges. When we encountered this we noted that the data were skewed and considered the implications.

#### 'Summary of findings' table

Two review authors (DGB and MF) assessed the certainty of the evidence (high, moderate, low, and very low) using the five GRADE considerations: risk of bias, inconsistency, imprecision, indirectness, and publication bias) (Guyatt 2008). We used methods and recommendations described in the *Cochrane Handbook for Systematic Reviews of Interventions* (Schünemann 2017) and the EPOC worksheets (EPOC 2017c), using GRADEpro software (GRADEpro GDT). We resolved disagreements on certainty ratings by discussion and provided justification for decisions to down‐ or upgrade the ratings using footnotes in the table, making comments to aid readers' understanding of the review where necessary. We used plain language statements to report these findings in the review (EPOC 2017d).

We created 'Summary of findings' tables for the following outcomes in order to draw conclusions about the certainty of the evidence within the text of the review:

- Providers' adherence to recommended practice, guidelines or protocols;
- Time between presentation and management of the health condition;
- Healthcare use;
- Participants' health status and well‐being;
- Participant and provider acceptability or satisfaction with the intervention;
- Costs;
- Technical problems.

We created three 'Summary of findings' tables, according to the setting where the intervention was delivered (primary, secondary and community care), as the populations in those settings, both healthcare providers and participants, are substantially different.

We considered whether there was any additional outcome information that we were not able to incorporate into meta‐analyses, noted this in the tables and stated whether it supports or contradicts the information from the meta‐analyses. When it was not possible to meta‐analyse the data, we summarised the results in the text and in the 'Comments' section of the 'Summary of findings' tables.

#### Subgroup analysis and investigation of heterogeneity

We categorised trials by setting (community, primary and secondary care), according to healthcare provider type, e.g. primary care doctors' or nurses' communication with hospital‐based specialists, or community health workers consulting with clinic staff.

We planned to use the following outcomes in subgroup analysis.

- Time between presentation and management of the health condition.
- Participants' health status and well‐being.

We planned to use the formal statistical techniques of Mantel‐Haenszel and regression to test for subgroup interactions (Mantel 1959) but due to the limited number of studies we could not use this technique.

#### Sensitivity analysis

We planned to perform sensitivity analyses defined a priori to assess the robustness of our conclusions and explore the impact on effect sizes. This would have involved restricting the analysis to published trials and to trials at low risk of bias. We did not perform sensitivity analyses as there were no unpublished trials and within the pooled analyses all the trials had the same risk of bias for the relevant 'Risk of bias' criteria.

## Results

### Description of studies

We identified 19 published randomised trials of mobile technologies to support healthcare provider to healthcare provider communication and management of care (see Characteristics of included studies).

#### Results of the search

We retrieved 20,949 records for title and abstract screening, screened the full‐text of 2041 citations and included 19 trials (35 citations) (Armstrong 2018; Azogil‐López 2019; Byamba 2015; Chang 2011; Davis 2003; Eminović 2009; Gulacti 2017; Iversen 2018; Liddy 2019a; Mansberger 2015; Orlandoni 2016; Pak 2007; Piette 2017; Riordan 2015; Sutherland 2009; Taylor‐Gjevre 2018; Van Gelder 2017; Whited 2002; Whited 2013). In addition, we identified 15 ongoing trials (ACTRN12617000389303; ACTRN12618001007224; Gervès‐Pinquié 2017; Jeandidier 2018; Källander 2015; Koch 2018; Nakayama 2016; Stevanovic 2017; NCT02821143; NCT02986256; NCT03137511; Done 2018; NCT03559712; NCT03662256; Xu 2017). A total of 441 records were eligible for the associated review on mobile technologies to support patient to healthcare provider communication and management of care (Gonçalves‐Bradley 2018a). Figure 1 presents the results of the search.

**1:**
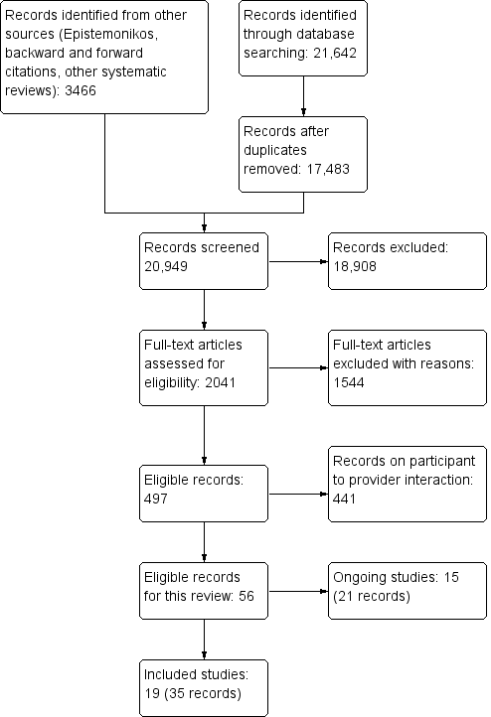
Flow diagram

#### Included studies

##### Trial populations

Seventeen trials included 5766 participants, while two trials did not report the specific number of participants (Liddy 2019a; Riordan 2015). The number of healthcare professionals recruited ranged from one general practitioner (GP) consulting with one ophthalmologist (Davis 2003), to another trial that randomised 113 GPs consulting with several specialty physicians (Liddy 2019a). Most of the trials involved primary care professionals consulting with specialists, namely dermatologists (Armstrong 2018; Byamba 2015; Eminović 2009; Pak 2007; Piette 2017; Whited 2002; Whited 2013), ophthalmologists (Davis 2003; Mansberger 2015), nephrologists (Van Gelder 2017) or radiologists (Sutherland 2009). In two studies more than one type of specialist was involved (Azogil‐López 2019; Liddy 2019a). The GPs mainly worked in urban settings and consulted with specialists also located in urban settings (N = 11). In four studies the GPs were located in rural settings, and consulted with providers in urban settings. There was one trial each for community‐based peer health workers consulting with clinic staff (Chang 2011), home‐visiting nursing staff consulting with a hospital physician (Orlandoni 2016), rural‐based physical therapists consulting with rheumatologists (Taylor‐Gjevre 2018), and community nurses consulting with specialist nurses or podiatrists (Iversen 2018). Two trials reported on emergency physicians consulting with hospital‐based specialists (Gulacti 2017; Riordan 2015).

All trials recruited adults, with Sutherland 2009 also recruiting adolescents and Azogil‐López 2019 recruiting participants aged seven years and older, and Orlandoni 2016 specifically recruiting participants aged 65 years and older. Three trials recruited participants with diabetes (Davis 2003; Mansberger 2015; Iversen 2018), and one with rheumatoid arthritis (Taylor‐Gjevre 2018). Seven trials recruited participants with a range of conditions seeking referral to a dermatologist (Armstrong 2018; Byamba 2015; Eminović 2009; Pak 2007; Piette 2017; Whited 2002; Whited 2013), two trials recruited participants attending the emergency department (Gulacti 2017; Riordan 2015) or requiring a hospital referral (Azogil‐López 2019; Liddy 2019a), and one trial each recruited participants requiring a trans‐abdominal or trans‐vaginal ultrasound (Sutherland 2009) or with chronic kidney disease (Van Gelder 2017). The two remaining trials recruited participants receiving antiretroviral therapy (Chang 2011) and home enteral nutrition (Orlandoni 2016).

##### Setting

Trials were mainly conducted in North America (9 trials) and Europe (six trials), with one trial each conducted in the Dominican Republic, Turkey, and Uganda, and Mongolia.

##### Interventions

The trials included in the review evaluated interventions that varied in mode of delivery, number of sessions, and healthcare providers involved. All trials used a portable device, 10 of them using a portable device to obtain clinical images which were then transmitted for further assessment (Armstrong 2018; Byamba 2015; Davis 2003; Eminović 2009; Mansberger 2015; Pak 2007; Piette 2017; Sutherland 2009; Whited 2002; Whited 2013). Four trials used mobile phones for text messages and voice calls (Chang 2011), secure messaging (Gulacti 2017), audio‐conferencing system (Azogil‐López 2019), and for interactive web‐based record and voice calls (Iversen 2018). Two trials used a tablet for secure messaging (Riordan 2015) or video consultation (Orlandoni 2016), whereas one trial employed a laptop for video consultation (Taylor‐Gjevre 2018). The remaining trials used an electronic health record system for eConsults, which could also be implemented through mobile phones (Liddy 2019a; Van Gelder 2017).

The trials also varied in the frequency and duration of contacts between the healthcare providers, with most trials consisting of a single consultation (e.g. Eminović 2009).

Although the control group was always described as receiving usual care, the description of the specific care received varied. For trials conducted in primary care, 'usual care' generally consisted of a referral for a face‐to‐face appointment in secondary care (Byamba 2015; Eminović 2009; Liddy 2019a; Pak 2007; Whited 2002; Whited 2013) or a reminder to book an appointment (Davis 2003; Mansberger 2015; Piette 2017; Sutherland 2009). For one trial that used a social media platform for emergency department physicians to communicate with specialists within the same hospital (Gulacti 2017), 'usual care' was to consult by phone, sending all clinical information verbally. For trials conducted in the community, 'usual care' was typically face‐to‐face appointments with specialists, either at the participant's home (Orlandoni 2016) or at outpatient clinics (Iversen 2018; Taylor‐Gjevre 2018).

Several trials reported on additional components of the intervention (Table 4). Nine reported the delivery of training (Armstrong 2018; Byamba 2015; Chang 2011; Eminović 2009; Iversen 2018; Mansberger 2015; Piette 2017; Sutherland 2009; Taylor‐Gjevre 2018), which usually focused on how to acquire digital images or use the web‐based system. For one trial of eConsult, the specialists received financial incentives for each eConsult they undertook (Liddy 2019a), and two trials provided monetary incentives for participants to take part (Armstrong 2018) or to complete follow‐up assessment (Mansberger 2015). Two trials reported that participants whose healthcare providers were allocated to the intervention group had increased access to health care, either directly (Armstrong 2018) or indirectly (Chang 2011).

**1:** Intervention components

**Study**	**Incentives**	**Specific training**
**Armstrong 2018**	Participants were paid for participating in the study, through gift cards (main paper, p.3, end 1st paragraph)	Participants and their carers were taught how to take standardised images of skin lesions, as well as how to communicate with the dermatologist using a secure web‐based system. PCPs also had access to the training materials. (Protocol, p.19, 2nd paragraph)
**Byamba 2015**	‐	GPs attended a 2‐day training session to learn how to take images and use the medical record system and software on mobile phones (p.1, top 2^nd^ column)
**Chang 2011**	PHWs were given a bicycle, t‐shirts, basic supplies, and an initial monthly allowance (parent trial)	PHWs allocated to the intervention group were given a mobile phone, and attended a 1‐day residential training and a brief field‐based practical training on the intervention (main paper, p.3, 2nd paragraph)
**Eminović 2009**	‐	GPs allocated to the intervention group received detailed instructions on how to take digital images and use the web‐based form (main paper, p.559, bottom 1st column)
**Iversen 2018**	‐	All staff received training in the use of the web‐based system, as well as in‐person access to hospital clinics to improve their practical skills (main paper, pp.97‐8)
**Liddy 2019a**	Specialists received financial incentives for each eConsult they undertook (support paper, under 8. Payment)	‐
**Mansberger 2015**	Participants received monetary incentive to complete follow‐up questionnaire (associated paper, p.524, bottom 1st column)	Technicians performing imaging attended a 3‐day training session to learn how to take images and ongoing feedback as needed (main paper, p.943, bottom 1st column)
**Piette 2017**	‐	GPs received training and a workbook on how to take photographs (p.2, top 2nd column)
**Sutherland 2009**	‐	The on‐site investigator received sonographic training over a 2‐month period, as well as practice guidelines for trans‐abdominal ultrasound scanning (P. 192, mid 1st column and top 2nd column)
**Taylor‐Gjevre 2018**	‐	Physical therapists and rheumatologists received an orientation and education session about rheumatoid arthritis and the study protocol and methods (main paper, p.2, top 2nd column)

GP: general practitioner; PCP: primary care provider; PHW: peer health workers

##### Funding, ethical approval, and conflict of interest

Sixteen trials reported funding sources, all of which were provided by medical research institutes or university funding bodies. One of the trials also received funding from a biopharmaceutical company (Van Gelder 2017). Three trials did not report ethical or institutional review board approval (Byamba 2015, letter; Davis 2003, short report; Riordan 2015, conference abstract).

For three trials one or more members of the author team reported financial support from pharmaceutical companies (Armstrong 2018, 3/29 authors; Van Gelder 2017 1/10 authors; Whited 2013, 1/18 authors). The lead author of Pak 2007 was the co‐founder of a web‐based consultation service identical to that used in the intervention. Six studies did not report conflicts of interest (Chang 2011; Davis 2003; Riordan 2015; Sutherland 2009; Taylor‐Gjevre 2018; Whited 2002), and for the remaining nine studies the authors had no known conflict of interest.

#### Excluded studies

We excluded 1544 full texts, of which we report on 22 excluded trials (See Characteristics of excluded studies). The most frequent reason for excluding trials was the explicit use of non‐mobile equipment (eight trials).

### Risk of bias in included studies

Figure 2 presents a graph for risk of bias and Figure 3 summarises risk of bias.

**2:**
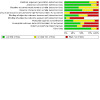
Risk of bias graph: review authors' judgements about each risk of bias item presented as percentages across all included studies.

**3:**
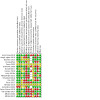
Risk of bias summary: review authors' judgements about each risk of bias item for each included study.

#### Allocation

Fourteen trials described the generation of the randomisation schedule, and were judged at low risk of bias (Armstrong 2018; Azogil‐López 2019; Byamba 2015; Eminović 2009; Gulacti 2017; Iversen 2018; Liddy 2019a; Mansberger 2015; Orlandoni 2016; Pak 2007; Piette 2017; Taylor‐Gjevre 2018; Van Gelder 2017; Whited 2013), one trial that 'tossed a coin' was judged as high risk of bias (Sutherland 2009), and we rated the remaining trials at unclear risk of bias. Fifteen trials were judged at low risk of bias for allocation concealment (Armstrong 2018; Azogil‐López 2019; Byamba 2015; Chang 2011; Eminović 2009; Iversen 2018; Liddy 2019a; Mansberger 2015; Orlandoni 2016; Pak 2007; Piette 2017; Taylor‐Gjevre 2018; Van Gelder 2017; Whited 2002; Whited 2013), one at high risk (Sutherland 2009), and the remaining trials were unclear due to a lack of information.

Eight trials reported baseline outcome measurements that were similar between groups, thus being assessed at low risk of bias (Armstrong 2018; Chang 2011; Eminović 2009; Liddy 2019a; Orlandoni 2016; Taylor‐Gjevre 2018; Whited 2002; Whited 2013), and the remaining 11 trials were assessed as being at unclear risk of bias. Ten trials reported similar baseline characteristics between groups and we judged them to be at low risk of bias (Armstrong 2018; Chang 2011; Eminović 2009; Gulacti 2017; Liddy 2019a; Mansberger 2015; Orlandoni 2016;Taylor‐Gjevre 2018; Van Gelder 2017; Whited 2013), three trials reported differences between groups at baseline and we judged them to be at high risk of bias (Azogil‐López 2019; Pak 2007; Piette 2017), and the remaining six trials were unclear.

#### Blinding

Due to the nature of the intervention it was often not possible to blind participants or healthcare professionals. We judged 16 trials to be at high risk of performance bias, and three at unclear (Byamba 2015; Davis 2003; Riordan 2015).

For objective outcomes we assessed six trials to be at high risk of detection bias (Mansberger 2015; Orlandoni 2016; Piette 2017; Taylor‐Gjevre 2018; Van Gelder 2017; Whited 2013), eight trials to be at low risk of bias and five trials to have an unclear risk of bias (Byamba 2015; Chang 2011; Davis 2003; Eminović 2009; Riordan 2015). For subjective outcomes we assessed eight trials to be at high risk of detection bias (Armstrong 2018; Eminović 2009; Iversen 2018; Piette 2017; Taylor‐Gjevre 2018; Van Gelder 2017; Whited 2002; Whited 2013), one trial to be at low risk of bias (Pak 2007), and two trials to have an unclear risk of bias (Davis 2003; Riordan 2015). Eight trials did not collect data on subjective outcomes.

#### Incomplete outcome data

Eight trials had high rates of incomplete outcome data and we judged them to be at high risk of attrition bias (Azogil‐López 2019; Chang 2011; Eminović 2009; Liddy 2019a; Orlandoni 2016; Pak 2007; Taylor‐Gjevre 2018; Whited 2013), and nine trials at low risk of attrition bias and were unclear about two trials (Davis 2003; Riordan 2015).

#### Selective reporting

We judged nine trials to be at high risk of reporting bias, as either outcomes were not reported per protocol (Armstrong 2018; Eminović 2009; Gulacti 2017; Iversen 2018; Taylor‐Gjevre 2018; Whited 2013) or publications were found for the same trial without cross‐reference (Chang 2011; Pak 2007; Whited 2002). For three trials it was not possible to make a judgement due to a lack of information (Davis 2003; Mansberger 2015; Riordan 2015), and seven trials had a low risk of reporting bias.

#### Other potential sources of bias

We judged other potential sources bias as unclear in three trials, two because there was not enough information (Davis 2003; Riordan 2015), and the other due to several methods being reported to collect outcome data due to problems with follow‐up (Eminović 2009). We judged one trial to have a high risk of other potential sources of bias, as data collection methods differed for the two trial groups and were not clearly reported (Mansberger 2015). There was no other apparent source of bias for the remaining trials and we judged them to be at a low risk of bias.

### Effects of interventions

See: Table 1; Table 2; Table 3

#### Comparison 1: Mobile technologies used by primary care providers to consult with hospital based specialists

Thirteen trials reported on mobile technologies used by primary care providers to consult with hospital‐based specialists. The studies involved GPs consulting with dermatologists (Armstrong 2018; Byamba 2015; Eminović 2009; Pak 2007; Piette 2017; Whited 2002; Whited 2013), ophthalmologists (Davis 2003; Mansberger 2015), radiologists (Sutherland 2009), nephrologists (Van Gelder 2017), or different specialists (Azogil‐López 2019; Liddy 2019a). The mobile component of the interventions consisted of a non‐mydriatic camera for retinal screening (Davis 2003; Mansberger 2015), portable ultrasound (Sutherland 2009), teledermatology to send digital images (Armstrong 2018; Byamba 2015; Eminović 2009; Pak 2007; Piette 2017; Whited 2002; Whited 2013), and eConsult through audio‐conferencing or secure direct messaging between healthcare providers, with a mobile component (Azogil‐López 2019;Liddy 2019a; Van Gelder 2017). For an overview of the evidence please refer to Table 1.

##### Main outcomes

###### 1. Providers' adherence to recommended practice, guidelines or protocols

One trial reported on the use of telenephrology by nephrologists to communicate with primary care providers for people with chronic kidney disease (CKD) (Van Gelder 2017). The authors found little or no difference for providers' adherence to the advised monitoring criteria from national CKD guidelines, as measured by monitoring of disease progression and metabolic parameters (3004 participants; moderate‐certainty evidence; Analysis 1.1). Follow‐up was not reported.

###### 2. Time between presentation and management of the health condition

Four trials reported on time between presentation and management of the health condition (656 participants; moderate‐certainty evidence; Analysis 2.1). Two trials recruited GPs who collected digital images from people with a skin condition and consulted with hospital‐based dermatologists on how to interpret them, reporting that people received the required treatment from their dermatologist in less time than those allocated to the control group: for Whited 2002 mean difference −40.5 days, 95% CI −23 to −58 days (275 participants); Piette 2017 reported a median of 4 days for the intervention group (IG) and 40 days for the control group (CG), with an adjusted hazard ratio (HR) of 2.55, P = 0.01 (103 participants). A third trial recruited GPs who shared ultrasound images with radiologists, finding little or no difference between groups on median time to participant follow‐up or diagnosis (Sutherland 2009; 105 participants). Azogil‐López 2019 recruited GPs who either referred their participants to an in‐person hospital appointment (control group) or to an audio‐consultation (intervention group), finding that those allocated to the audio‐consultation waited for less time (median −27 days, 99% CI −20 to −33 days; 173 participants). Follow‐up, when provided, ranged between three and six months.

##### Other outcomes

###### 1. Healthcare use

Nine trials reported on various forms of healthcare use, including referrals, screening examinations, outpatient visits and hospitalisations (4810 participants; moderate‐certainty evidence; Analysis 3.1).

Four trials recruited GPs who consulted with dermatologists through the use of digital images (Byamba 2015; Piette 2017; Whited 2002; Whited 2013; 4 trials, 1075 participants; follow‐up between three and nine months, when reported), finding that those participants allocated to the intervention group were less likely to subsequently receive a referral for an appointment with a dermatologist, visit a dermatology clinic, or be referred to tertiary care: risk ratio (RR) ranged from 0.28 (95% CI 0.21 to 0.38) to 0.82 (0.75 to 0.88). We did not retain the meta‐analysis because of high statistical heterogeneity (Analysis 3.2; I^2^ = 91%).

One trial of eConsults between PCPs and nephrologists reported that there was little or no difference between groups for referral rate (odds ratio (OR) 0.61, 95% CI 0.31 to 1.23; Van Gelder 2017; 3004 participants). Another trial of eConsults between PCPs and a range of specialists also found little or no difference between groups for face‐to‐face referral (RR 0.93, 95% CI 0.85 to 1.03; Liddy 2019a).

Two trials of retinopathy screening for participants with diabetes (Davis 2003; Mansberger 2015) reported that those allocated to the intervention group were more likely to receive a screening examination (2 trials, 626 participants; 12 months follow‐up when reported). High statistical heterogeneity precluded retaining the meta‐analysis (Analysis 3.3; I^2^ = 85%). Another trial of GPs consulting with radiologists about participants requiring a trans‐abdominal or trans‐vaginal ultrasound found that participants allocated to the intervention group were more likely to receive an ultrasound (RR 3.92, 95% CI 2.11 to 7.31; Sutherland 2009; 105 participants).

###### 2. Participants' health status and well‐being

Two trials reported on a dermatologist providing feedback to GPs based on digital images, finding similar scores between those allocated to the intervention and the control group, for general health status at 12‐month follow‐up (Armstrong 2018), as well as quality of life and health‐related quality of life as reported by the participants, at nine‐month follow‐up (Whited 2013) (2 trials, 622 participants; moderate‐certainty evidence; Analysis 4.1). Two teledermatology trials reported on clinical course as assessed by dermatologists at four‐ (Pak 2007) and nine‐month follow‐up (Whited 2013), finding little or no difference between groups in clinical course (2 trials, 769 participants; moderate‐certainty evidence; Analysis 4.2).

###### 3. Healthcare provider acceptability and satisfaction

Two trials (378 participants) recruited GPs who consulted with dermatologists using digital images (low‐certainty evidence); Piette 2017 reported little or no difference between groups for acceptability or satisfaction, and Whited 2002 reported that GPs allocated to the intervention were more likely to agree that participants received timely appointments and to be satisfied with the consult process than GPs allocated to the control group. One additional trial (Van Gelder 2017) reported on satisfaction for healthcare professionals allocated to the intervention group (Analysis 5.1).

###### 4. Participant acceptability and satisfaction

Four trials (972 participants, low‐certainty evidence; Analysis 5.2) recruiting GPs who consulted with dermatologists through the use of digital images reported little or no difference in participant satisfaction between those allocated to the intervention or to care as usual (Eminović 2009; Piette 2017; Whited 2002; Whited 2013).

###### 5. Costs

Six trials reported costs (5423 participants; low‐certainty evidence; Analysis 6.1). One teledermatology trial reported that the expected cost per participant per visit was higher for the intervention group (Whited 2002; 275 participants); a second teledermatology trial reported that the total direct costs were lower for the comparison group (Pak 2007; 698 participants; MD USD −4678, 95% CI −4720 to −4635), and that this difference was offset by the lost productivity for participants allocated to the control group (MD USD 14,409, 95% CI 14,398 to 14,419). Another teledermatology trial reported little or no difference between groups for total costs per participant from the healthcare perspective (MD USD 30, 95% CI USD −79 to 20), and from the societal perspective that included the cost of loss of productivity (MD USD −82, 95% CI −12 to −152) per participant allocated to the intervention (Whited 2013; 391 participants). Two trials (teledermatology and telenephrology, respectively) reported little or no difference between groups for costs (Eminović 2009, 605 participants; MD EUR 32.5, 95% CI −29.0 to 74.7; Van Gelder 2017; 3004 participants; IG: EUR 453.86, 95% CI 392.98 to 514.74; CG EUR 433.74, 95% CI 387.64 to 479.84, P = 0.60). One teledermatology trial set in rural areas in Mongolia reported lower costs associated with the intervention group, mainly explained by the long distances that those allocated to the control group had to travel, which was avoided with teledermatology (Byamba 2015; 450 participants, IG: USD 320, CG: 3174, difference USD 2854).

###### 6. Unintended consequences

Four trials reported on the quality of the data transmitted (Analysis 7.1). However, only one trial recruiting GPs consulting with dermatologists about images they took from their participants reported data for both groups (Pak 2007), reporting that 10 images from each group were lost due to technical problems (1 trial, 698 participants; moderate‐certainty evidence). The remaining trials reported results for the intervention group only (Piette 2017, Sutherland 2009, Whited 2002).

One trial where GPs could consult with dermatologists about people with psoriasis collected data about mortality as part of adverse events, reporting one death for each group (IG: 1/148; CG: 1/148; Armstrong 2018).

#### Comparison 2: Mobile technologies for communication between specialists in the emergency department

Two trials reported on mobile technologies for communication between physicians and specialists in the emergency department (Gulacti 2017; Riordan 2015), using a smartphone application for secure messaging. For an overview of the evidence please refer to Table 2.

##### Main outcomes

###### 1. Providers' adherence to recommended practice, guidelines or protocols

Neither of the trials of mobile technologies for communication between specialists in the emergency department reported data on providers' adherence.

###### 2. Time between presentation and management of the health condition

One trial that recruited emergency physicians who consulted with specialist physicians using a smartphone application reported that participants allocated to the intervention group were probably either admitted to hospital or discharged in slightly less time from the emergency department (median difference −12 minutes, 95% CI −19 to −7; 345 participants; moderate‐certainty evidence) (Gulacti 2017; Analysis 8.1).

##### Other outcomes

###### 1. Healthcare use

One trial reported that participants seen by emergency physicians allocated to the intervention group probably had a shorter length of emergency department stay (median difference −30 minutes, 95% CI −37 to −25 minutes; 345 participants; moderate‐certainty evidence; Analysis 9.1, Gulacti 2017).

###### 2. Participants' health status and well‐being

Neither of the trials of mobile technologies for communication between specialists in the emergency department reported data on participants' health status and well‐being.

###### 3. Healthcare provider acceptability and satisfaction

Neither of the trials on mobile technologies for communication between specialists in the emergency department reported data on healthcare provider acceptability or satisfaction.

###### 4. Participant acceptability and satisfaction

Neither of the trials on mobile technologies for communication between specialists in the emergency department reported on participant acceptability and satisfaction.

###### 5. Costs

Neither of the trials on mobile technologies for communication between specialists in the emergency department reported data on costs.

###### 6. Unintended consequences

Gulacti 2017 reported that there were no technical problems during the course of the trial (Analysis 10.1).

#### Comparison 3: Mobile technologies used by community health or home‐care workers

Four trials reported on mobile technologies used by community‐based health workers or home‐care workers. The professionals involved were community‐based peer health workers consulting with clinic staff about receiving antiretroviral therapy (Chang 2011); community nurses consulting with diabetes specialist nurses or podiatrists about adults with Type 2 diabetes (Iversen 2018); home‐care nurses consulting with hospital specialists about home enteral nutrition (Orlandoni 2016); and rural‐based physical therapists consulting with urban‐based rheumatologists (Taylor‐Gjevre 2018). The mobile‐based component of the interventions consisted of mobile phone, teledermatology, video‐consultations, and interactive web‐based records, respectively. For an overview of the evidence please refer to Table 3.

##### Main outcomes

###### 1. Providers' adherence to recommended practice, guidelines or protocols

None of the trials of mobile technologies used by community‐based health workers reported data on providers' adherence.

###### 2. Time between presentation and management of the health condition

None of the trials of mobile technologies used by community health workers reported data on time between presentation and management of the health condition.

##### Other outcomes

###### 1. Healthcare use

Two studies reported on outpatient clinic and community nurse consultations (370 participants, moderate‐certainty evidence). Iversen 2018 recruited community nurses consulting with diabetes specialist nurses and podiatrists about adults with new diabetes‐related foot ulcers, reporting little or no difference between groups for outpatient consultations (0.48 fewer consultations in the intervention group, 95% CI −1.46 to 0.49) or community nurse consultations (0.92 more consultations in the intervention group, 95% CI −0.70 to 2.53). One trial (188 participants) that recruited home‐visiting staff who consulted with hospital physicians through video‐conferencing about older adults treated with home enteral nutrition reported little or no difference for healthcare use, as measured by outpatient visits (Incidence rate ratio 95% CI 0.65 to 1.30, P = 0.62) and hospitalisations (Incidence rate ratio 95% CI 0.54 to 1.19, P = 0.26) (Orlandoni 2016; low‐certainty evidence; Analysis 11.1).

###### 2. Participants' health status and well‐being

Two trials, one recruiting community‐based peer health workers consulting with clinic staff about adults who were receiving or started receiving antiretroviral therapy (Chang 2011) and another recruiting community nurses consulting with diabetes specialist nurses and podiatrists about adults with new diabetes‐related foot ulcers (Iversen 2018), reported mortality at 11‐ to 12‐month follow‐up (RR 0.82, 95% CI 0.55 to 1.22 and RR 0.94, 95% CI 0.28 to 3.12, respectively; 1157 participants; low‐certainty evidence; Analysis 12.1).

One trial (85 participants) of rural‐based physical therapists consulting with urban‐based rheumatologists about adults with a clinical diagnosis of rheumatoid arthritis reported little or no difference between groups for health‐related quality of life and disease activity (low‐certainty evidence) (Taylor‐Gjevre 2018; Analysis 12.1).

###### 3. Healthcare provider acceptability and satisfaction

None of the trials of mobile technologies used by community health workers reported data on healthcare provider acceptability or satisfaction.

###### 4. Participant acceptability and satisfaction

Two trials (178 participants) reported on participants' experience with healthcare (Iversen 2018) and satisfaction with the intervention (Taylor‐Gjevre 2018), reporting little or no difference between those allocated to the intervention or the control groups (moderate‐certainty evidence) (Analysis 13.1).

###### 5. Costs

One trial reported the total cost of running the intervention and cost per participant for the intervention group only (Chang 2011, Analysis 14.1).

###### 6. Unintended consequences

A trial that recruited community‐based peer health workers consulting with clinic staff about adults who were receiving or started receiving antiretroviral therapy reported that healthcare professionals allocated to the intervention were not always able to charge the mobile phone, and that some mobile phones were stolen (Chang 2011; Analysis 15.1). Another trial where community nurses consulted with diabetes specialist nurses and podiatrists about adults with new diabetes‐related foot ulcers through videoconference, reported that images were not always transmitted (Taylor‐Gjevre 2018).

#### Equity considerations

Some of the included trials were designed and implemented to address geographical (Byamba 2015; Chang 2011; Davis 2003; Taylor‐Gjevre 2018) or socio‐economic limitations (Mansberger 2015; Sutherland 2009) on access to health care, and thus to promote equity for rural‐based and other disadvantaged populations who would have less access to health care (Table 5).

**2:** Equity considerations

**Study ID**	**Population**	**Disadvantaged populations included/excluded?**	**Notes**
**Armstrong 2018**	General practitioner consulting with dermatologists about adults with psoriasis	Participants without access to the Internet and a digital camera or smartphone with camera features were excluded	‐
**Azogil‐López 2019**	GP consulting with hospital physicians about participants (aged ≥ 7 years)	Participants deemed as complex were not eligible for receiving the intervention	Complex participants defined as those lacking a specific diagnosis or requiring further clinical assessment
**Byamba 2015**	GP consulting with dermatologists about adults with skin lesions	Intervention was set in rural health clinics in Mongolia	‐
**Chang 2011**	Community‐based peer health workers consulting with clinic staff about adults who were receiving or started receiving antiretroviral therapy	Specifically targeted HIV‐positive participants in rural Uganda. However, many participants had limited access to mobile phones*, which might have limited the benefits of the intervention. For the healthcare providers, the costs of the intervention were also a factor, as although they were given a monthly stipend it was not always enough Charging the mobile phone was often challenging, as access to electricity was limited	* Current mobile phone penetration in Uganda at the time the trial was conducted was 39%
**Davis 2003**	PCPs at the rural primary practice consulting with ophthalmologist in the university setting about adults with Type 2 diabetes	Specifically targeted rural‐based ethnic minorities, 35% of whom did not have health insurance	‐
**Gulacti 2017**	Emergency physicians consulting with specialists about adults attending the emergency department	Only consultants who owned a smartphone and were familiarised with the secure messaging service were included	‐
**Mansberger 2015**	PCPs consulting with experienced investigators based at an eye institute about adults with Type 2 diabetes	Primary clinics that served a large number of ethnic minorities, including a high percentage of participants with transient housing	‐
**Piette 2017**	General practitioners consulting with dermatologists about adults with skin lesions	Participants who were not able to attend in‐person appointments at the dermatologist office were excluded, i.e. participants unable to travel or those residing in nursing homes.	‐
**Sutherland 2009**	GP consulting with radiologists about participants aged ≥ 13 years requiring a trans‐abdominal or trans‐vaginal ultrasound	Sample was composed mainly of low‐skilled workers relying on government‐supported primary clinics for their health care	‐
**Taylor‐Gjevre 2018**	Community nurses consulting with diabetes specialist nurses and podiatrists about adults aged ≥ 20 years with new diabetes‐related foot ulcers	Specifically targeted rural‐based adults	‐
**Whited 2013**	GP consulting with dermatologists about adults with skin condition	Participants who could not speak or read English or who failed a single‐question literacy assessment* were excluded	*Single‐Item Literacy Screener (SILS), which identifies limited reading ability (Morris 2006)

GP: General practitioner; PCP: primary care provider; PHW: Peer health workers

Even when a trial was specifically designed to address inequities identified a priori, it might still exclude the most vulnerable elements of the targeted population. Chang 2011 recruited peer health workers in rural Uganda, giving them access to experienced clinical staff through text messages and mobile phone calls, in order to provide better health care to HIV‐positive people. The peer health workers could interact with the participants using the mobile phone. The authors concluded that the relatively low penetration of mobile phones in Uganda, which at the time was 39%, alongside the challenges posed with phone‐charging in a setting where access to electricity is limited, might not only have limited the benefits of the intervention but also increased inequities. Furthermore, the authors also noted that the costs of the intervention could have been a limiting factor for the peer health workers, as the monthly stipend given for mobile phone credits was not always enough.

Whited 2013 excluded people who could not speak or read English, as well as those who failed a single‐question literacy assessment. Gulacti 2017 assessed the use of a messaging system for communication between emergency physicians in the emergency department and physicians working elsewhere in the hospital, excluding consultants who did not own a smartphone with a secure messaging service. Armstrong 2018 excluded people without access to the Internet and either a digital camera or a mobile phone with camera features.

## Discussion

### Summary of main results

We included 19 randomised trials of mobile technologies that recruited more than 5766 participants with varied conditions and health problems. Healthcare professionals included general practitioners, community‐based peer health workers, nurses and physiotherapists, who consulted with specialist healthcare professionals in another healthcare facility, and emergency physicians who consulted colleagues within the same facility. Most trials reported on the use of mobile technologies by general practitioners to consult with specialists, and reported that mobile technologies reduced the time between presentation and management of the health problem (4 trials, 656 participants; moderate‐certainty evidence). Accessing healthcare services through mobile technologies may reduce referrals and clinic visits among people with skin conditions and those with chronic kidney disease, and increase the likelihood of receiving an eye examination among people with diabetes and people referred for an ultrasound (9 trials, 4810 participants when reported, moderate‐certainty evidence). There was little evidence of a difference to patient‐reported quality of life outcomes (2 trials, 622 participants), clinician‐reported outcomes of disease progression (2 trials, 769 participants); or to healthcare providers and participants' satisfaction and acceptability, or cost (6 trials, 5423 participants, low‐certainty evidence). One trial reported on images being lost during transmission, when using mobile technologies and also in usual care; and one trial reported a few experiences of mobile phones not being charged or being lost. However, most trials did not measure or report technical problems.

Four studies reported on the use of mobile technologies by community health or home‐care workers to consult with clinic staff, there was little evidence of an effect on consultations in the trials that recruited participants with new diabetes‐related foot ulcer or older individuals treated with home enteral nutrition (2 trials, 370 participants; moderate‐certainty evidence). There was little or no difference for hospitalisations among older individuals treated with home enteral nutrition (1 trial, 188 participants; low‐certainty evidence), or mortality among people living with HIV or diabetes (2 trials, 1157 participants), for disease activity and health‐related quality of life in participants with rheumatoid arthritis (1 trial, 85 participants) or participant acceptability and satisfaction in people with new diabetes‐related foot ulcer or rheumatoid arthritis (178 participants).

### Overall completeness and applicability of evidence

Most trials did not report data on providers' adherence, five trials reported on time between presentation and management of the health condition for the main comparison, and for the remaining comparisons and outcomes we identified very little evidence. A third of the trials recruited adults seeking care for dermatological conditions, reflecting current use of mobile technologies in healthcare settings.

The use of mobile technologies for communication between healthcare professionals and patient management might be particularly relevant for settings where there is a shortage of healthcare providers. However, most of the trials were conducted in high‐income (eleven trials in North America and six trials in Europe) or upper‐middle‐income countries (two trials, one in North America and one in Asia), with one trial each conducted in a lower‐middle rural country (Mongolia) and a low‐income country (Uganda). A similar range of countries was reported in a review of mobile technologies for healthcare service delivery processes (Free 2013). Specific challenges might arise when implementing trials in those contexts, such as the lack of access to power sockets to charge the mobile phones, highlighted by the peer health workers interviewed by Chang 2011 in Uganda but not in the study conducted in Mongolia.

Similar contextual factors might contribute to the applicability of the evidence. One factor often mentioned was the variation in healthcare professionals' willingness to use mHealth (Azogil‐López 2019; Liddy 2019a), to attend training (Liddy 2019a), to invite people to participate (Eminović 2009), to select participants for electronic referrals (Van Gelder 2017), or to hold face‐to‐face appointments (Iversen 2018), or provide the required feedback (Sutherland 2009). Four trials reported that recruited participants might not have been representative of the general population, as the study population was more educated (Armstrong 2018), more likely to be male (Mansberger 2015; Whited 2002; Whited 2013), and more likely to be healthier (Armstrong 2018; Mansberger 2015). There was also variation associated with participants' location, as those allocated to the intervention who lived closer to the referral setting were less likely to accept a telephone appointment (Azogil‐López 2019) and more likely to be referred to a face‐to‐face appointment from their healthcare provider (Iversen 2018). One trial reported that participants allocated to the control group had to travel on average 98 km to receive face‐to‐face care, thus indicating that the intervention might provide particular benefits in settings with a low‐density population (Byamba 2015).

Some of the included trials were designed and implemented to address geographical limitations on access to health care and thus allowed for healthcare providers who were geographically separated to exchange clinical information, promoting equity for rural‐based and other disadvantaged populations who would have less access to healthcare. Two trials were conducted in rural settings: South Carolina, USA (Davis 2003) and Saskatchewan, Canada (Taylor‐Gjevre 2018). Two trials recruited participants from socio‐economically disadvantaged areas (Mansberger 2015; Sutherland 2009).

#### Certainty of the evidence

The included randomised trials were mostly at low or unclear risk of selection bias. We downgraded the evidence for almost all of the outcomes due to a high risk of performance bias, and almost half of the trials were also at risk of detection, attrition, and reporting biases. We also downgraded some of the evidence for imprecision, due to the relatively small size of the trials. Our confidence in the effect estimates overall is moderate, although due to the relatively low number of trials, different uses of mHealth interventions and small numbers of participants recruited there is a possibility that the estimate of the effect is substantially different.

### Potential biases in the review process

We limited the risk of publication bias by conducting a comprehensive literature search of different databases, including published articles, clinical trials registries and unpublished mHealth evidence. The WHO issued a call for papers through popular digital health communities of practice to identify additional primary trials as well as grey literature, all of which have contributed to limit publication bias. Two review authors screened records, extracted data and assessed the certainty of the evidence using GRADE, with discussion with the author team whenever there were any discrepancies.

### Agreements and disagreements with other studies or reviews

Hasselberg 2014 conducted a review on image‐based medical expert teleconsultation, with 24 studies, including non‐randomised and feasibility studies. The overall results were similar to ours. A review on asynchronous electronic consultations that included 36 trials, seven of which were randomised trials, reported that healthcare providers were generally satisfied with the timely advice received and the health care provided to the participants (Liddy 2016). When updating the review, Liddy 2019b included non‐randomised evidence and concluded that eConsults were expanding beyond teledermatology and that providers from other specialties were also satisfied. We found limited evidence from randomised trials about how satisfied healthcare providers are with mHealth to communicate with other providers. For both reviews the authors concluded that there was limited research on morbidity and mortality, which is consistent with our results (Liddy 2016; Liddy 2019b).

A Cochrane qualitative evidence synthesis (QES) on healthcare providers' perceptions and experiences of using mHealth technologies to deliver primary care healthcare services found that while providers thought that mobile technologies improved their work and relationships with other providers as well as participants, they also highlighted specific challenges such as access to electricity and network coverage (Odendaal 2020). Similarly, an unpublished overview of factors influencing the acceptability, feasibility and implementation of mobile health technologies also reported problems with installation and usability, as well as issues with electricity and connection (Glenton 2019). This is consistent with our results, especially for settings where constant access to electricity might be an issue (Chang 2011).

## Authors' conclusions

**Implications for practice**

Mobile technologies are widespread, with the quality of transmission continuing to improve. Healthcare organisations in a number of settings have started to provide their healthcare providers with smartphones (Dala‐Ali 2011) and healthcare professionals often use their mobile phones to share clinical information, including the transmission of images (Mobasheri 2015). This review found that mobile technologies may reduce the time between presentation and management of the health condition when primary care providers or emergency physicians use them to consult with specialists, may increase the likelihood of receiving a clinical examination among participants with diabetes and those who required an ultrasound and may reduce referrals to secondary or tertiary care.

One concern that has been raised is about data‐sharing and privacy (Chang 2011; Gulacti 2017; WHO 2011). Most of the included trials reported using secure web connections, and mobile phone applications are being developed for secure communications between medical staff at work. A recent review reported that the main barriers to the adoption of mHealth by healthcare professionals concern the perceived usefulness and ease of use, concerns surrounding privacy, security, and technological issues, cost, time, and how it will impact the interaction with colleagues, patients, and management (Gagnon 2016), even in areas where the use of mobile technologies is more common. Training is usually required to support implementation, for instance teledermatology has been implemented in several settings and its optimal implementation includes training of primary healthcare providers on how to use the mobile equipment to obtain high‐quality images (Kukutsch 2017); this was highlighted by some of the included trials (e.g. Eminović 2009; Piette 2017).

There was little evidence about healthcare providers' satisfaction with the intervention in the trials we identified, and although healthcare providers reported that mobile technologies allowed for care to be delivered more quickly and facilitated triage, one study reported that they were less confident in their diagnosis and management plans when using teledermatology, compared with face‐to‐face care (Whited 2002). However, it is likely that this would improve with experience. A qualitative evidence synthesis reported that mobile technologies assisted contact with colleagues, and recommended that healthcare providers should be part of the planning, implementation, and evaluation of mobile health programmes. (Odendaal 2020). Similarly, it is important to establish whether mobile devices alleviate providers' workload, or instead add to it, including whether there is the capacity to provide the level of supervision and support required (Odendaal 2020).

**Implications for research**

- Funding is required to support the conduct of randomised trials of mobile technology interventions in settings where these types of intervention may have the potential to significantly strengthen health systems, such as remote locations and where there is a shortage of specialist services.
- Process evaluations, conducted alongside randomised trials, to identify factors that might modify the effect of mHealth interventions in different contexts would be a valuable addition to the evidence base (Craig 2008). Identifying core outcomes might be a useful step, for example, understanding the impact of mHealth on providers' adherence to guidelines, time from presentation to resolution, and participants' health status and well‐being are outcomes for which more evidence is required. Research should also be conducted into consideration of factors to support implementation, such as the high attrition rates commonly found in studies that use mobile technologies.
- Detailed and standardised reporting of mobile health interventions, technical features and context will contribute to the quality of the evidence available (Agarwal 2016).

## History

Protocol first published: Issue 1, 2018 Review first published: Issue 8, 2020

## Notes

This review is based on standard text and guidance provided by Cochrane Effective Practice and Organisation of Care (EPOC).

## Appendices

### Appendix 1. Search strategies and results

**Ovid MEDLINE(R) In‐Process & Other Non‐Indexed Citations, Ovid MEDLINE(R) Daily and Ovid MEDLINE(R) <1946 to Present> ‐ Searched 25 September 2018**

**Table:**

1	exp Health Personnel/
2	(((health or medical or healthcare) adj (personnel or worker* or auxiliar* or staff or professional*)) or doctor* or physician* or GP or general practitioner? or family doctor or nurse* or midwi* or clinical officer* or pharmacist* or dentist* or ((birth or childbirth or labor or labour) adj (attendant? or assistant?))).ti,ab,kw.
3	((lay or voluntary or volunteer? or untrained or unlicensed or nonprofessional? or non professional?) adj5 (worker? or visitor? or attendant? or aide or aides or support$ or person$ or helper? or carer? or caregiver? or care giver? or consultant? or assistant? or staff)).ti,ab,kw.
4	(paraprofessional? or paramedic or paramedics or paramedical worker? or paramedical personnel or allied health personnel or allied health worker? or support worker? or home health aide?).ti,ab,kw.
5	((community or village? or lay) adj3 (health worker? or health care worker? or healthcare worker?)).ti,ab,kw.
6	(doula? or douladural? or barefoot doctor?).ti,ab,kw.
7	1 or 2 or 3 or 4 or 5 or 6
8	Cell Phones/
9	Smartphone/
10	MP3‐Player/
11	Computers, Handheld/
12	((cell* or mobile*) adj1 (phone* or telephone* or technolog* or device*)).ti,ab,kw.
13	(handheld or hand‐held).ti,ab,kw.
14	(smartphone* or smart‐phone* or cellphone* or mobiles).ti,ab,kw.
15	((personal adj1 digital) or (PDA adj3 (device* or assistant*)) or MP3 player* or MP4 player*).ti,ab,kw.
16	(samsung or nokia).ti,ab,kw.
17	(windows adj3 (mobile* or phone*)).ti,ab,kw.
18	android.ti,ab,kw.
19	(ipad* or i‐pad* or ipod* or i‐pod* or iphone* or i‐phone*).ti,ab,kw.
20	(tablet* adj3 (device* or computer*)).ti,ab,kw.
21	Telemedicine/
22	Videoconferencing/ or Webcasts as topic/
23	Text Messaging/
24	Telenursing/
25	(mhealth or m‐health or "mobile health" or ehealth or e‐health or "electronic health").ti,ab,kw.
26	(telemedicine or tele‐medicine or telehealth or tele‐health or telecare or tele‐care or telenursing or tele‐nursing or telepsychiatry or tele‐psychiatry or telemonitor* or tele‐monitor* or teleconsult* or tele‐consult* or telecounsel* or tele‐counsel* or telecoach* or tele‐coach*).ti,ab,kw.
27	(videoconferenc* or video‐conferenc* or webcast* or web‐cast*).ti,ab,kw.
28	(((text* or short or voice or multimedia or multi‐media or electronic or instant) adj1 messag*) or instant messenger).ti,ab,kw.
29	(texting or texted or texter* or ((sms or mms) adj (service* or messag*)) or interactive voice response* or IVR or voice call* or callback* or voice over internet or VOIP).ti,ab,kw.
30	(Facebook or Twitter or Whatsapp* or Skyp* or YouTube or "You Tube" or Google Hangout*).ti,ab,kw.
31	Mobile Applications/
32	"mobile app*".ti,ab,kw.
33	Social Media/
34	(social adj (media or network*)).ti,ab,kw.
35	Reminder Systems/
36	(remind* adj3 (text* or system* or messag*)).ti,ab,kw.
37	Electronic Mail/
38	(electronic mail* or email* or e‐mail or webmail).ti,ab,kw.
39	Medical informatics/ or Medical informatics applications/
40	Nursing informatics/ or Public health informatics/
41	((medical or clinical or health or healthcare or nurs*) adj3 informatics).ti,ab,kw.
42	Multimedia/
43	Hypermedia/
44	Blogging/
45	(multimedia or multi‐media or hypermedia or hyper‐media or blog* or vlog* or weblog* or web‐log*).ti,ab,kw.
46	Interactive Tutorial/
47	Computer‐Assisted Instruction/
48	((interactive or computer‐assisted) adj1 (tutor* or technolog* or learn* or instruct* or software or communication)).ti,ab,kw.
49	or/8‐48
50	randomized controlled trial.pt.
51	controlled clinical trial.pt.
52	randomized.ab.
53	placebo.ab.
54	drug therapy.fs.
55	randomly.ab.
56	trial.ab.
57	groups.ab.
58	or/50‐57
59	exp animals/ not humans.sh
60	58 not 59
61	7 and 49 and 60
62	limit 61 to yr="2000 ‐Current"

**Embase (Ovid) ‐ Searched 25 September 2018**

**Table:**

1	mobile phone/ or smartphone/
2	mp3 player/
3	((cell* or mobile*) adj1 (phone* or telephone* or technolog* or device*)).ti,ab,kw.
4	(handheld or hand‐held).ti,ab,kw.
5	(smartphone* or smart‐phone* or cellphone* or mobiles).ti,ab,kw.
6	((personal adj1 digital) or (PDA adj3 (device* or assistant*)) or MP3 player* or MP4 player*).ti,ab,kw.
7	(samsung or nokia).ti,ab,kw.
8	(windows adj3 (mobile* or phone*)).ti,ab,kw.
9	android.ti,ab,kw.
10	(ipad* or i‐pad* or ipod* or i‐pod* or iphone* or i‐phone*).ti,ab,kw.
11	(tablet* adj3 (device* or computer*)).ti,ab,kw.
12	telemedicine/ or telecardiology/ or teleconsultation/ or teledermatology/ or telediagnosis/ or telemonitoring/ or telepathology/ or telepsychiatry/ or teleradiotherapy/ or telesurgery/ or teletherapy/
13	videoconferencing/ or webcast/
14	text messaging/
15	telenursing/
16	(mhealth or m‐health or "mobile health" or ehealth or e‐health or "electronic health").ti,ab,kw.
17	(telemedicine or tele‐medicine or telehealth or tele‐health or telecare or tele‐care or telenursing or tele‐nursing or telepsychiatry or tele‐psychiatry or telemonitor* or tele‐monitor* or teleconsult* or tele‐consult* or telecounsel* or tele‐counsel* or telecoach* or tele‐coach*).ti,ab,kw.
18	(videoconferenc* or video‐conferenc* or webcast* or web‐cast*).ti,ab,kw.
19	(((text* or short or voice or multimedia or multi‐media or electronic or instant) adj1 messag*) or instant messenger).ti,ab,kw.
20	(texting or texted or texter* or ((sms or mms) adj (service* or messag*)) or interactive voice response* or IVR or voice call* or callback* or voice over internet or VOIP).ti,ab,kw.
21	(Facebook or Twitter or Whatsapp* or Skyp* or YouTube or "You Tube" or Google Hangout*).ti,ab,kw.
22	mobile application/
23	"mobile app*".ti,ab,kw.
24	social media/
25	(social adj (media or network*)).ti,ab,kw.
26	reminder system/
27	(remind* adj3 (text* or system* or messag*)).ti,ab,kw.
28	e‐mail/
29	(electronic mail* or email* or e‐mail or webmail).ti,ab,kw.
30	medical informatics/
31	nursing informatics/
32	((medical or clinical or health or healthcare or nurs*) adj3 informatics).ti,ab,kw.
33	multimedia/
34	hypermedia/
35	blogging/
36	(multimedia or multi‐media or hypermedia or hyper‐media or blog* or vlog* or weblog* or web‐log*).ti,ab,kw.
37	teaching/
38	((interactive or computer‐assisted) adj1 (tutor* or technolog* or learn* or instruct* or software or communication)).ti,ab,kw.
39	or/1‐38
40	exp health care personnel/
41	(((health or medical or healthcare) adj (personnel or worker* or auxiliar* or staff or professional*)) or doctor* or physician* or GP or general practitioner? or family doctor or nurse* or midwi* or clinical officer* or pharmacist* or dentist* or ((birth or childbirth or labor or labour) adj (attendant? or assistant?))).ti,ab,kw.
42	((lay or voluntary or volunteer? or untrained or unlicensed or nonprofessional? or non professional?) adj5 (worker? or visitor? or attendant? or aide or aides or support$ or person$ or helper? or carer? or caregiver? or care giver? or consultant? or assistant? or staff)).ti,ab,kw.
43	(paraprofessional? or paramedic or paramedics or paramedical worker? or paramedical personnel or allied health personnel or allied health worker? or support worker? or home health aide?).ti,ab,kw.
44	((community or village? or lay) adj3 (health worker? or health care worker? or healthcare worker?)).ti,ab,kw.
45	(doula? or douladural? or barefoot doctor?).ti,ab,kw.
46	or/40‐45
47	39 and 46
48	crossover procedure/
49	double blind procedure/
50	randomized controlled trial/
51	single‐blind procedure/
52	random$.tw.
53	factorial$.tw.
54	(crossover$ or cross over$ or cross‐over$).tw.
55	placebo$.tw.
56	(doubl$ adj blind$).tw.
57	(singl$ adj blind$).tw.
58	assign$.tw.
59	allocat$.tw.
60	volunteer$.tw.
61	or/48‐60
62	47 and 61
63	limit 62 to yr="2000 ‐Current"
64	limit 63 to embase

**CENTRAL Register of Trials (Cochrane Library) – Searched 25 September 2018**

**Table:**

#1	MeSH descriptor: [Health Personnel] explode all trees
#2	(((health or medical or healthcare) near (personnel or worker* or auxiliar* or staff or professional*)) or doctor* or physician* or GP or general practitioner? or family doctor or nurse* or midwi* or clinical officer* or pharmacist* or dentist* or ((birth or childbirth or labor or labour) near (attendant? or assistant?))):ti,ab,kw
#3	((lay or voluntary or volunteer? or untrained or unlicensed or nonprofessional? or non professional?) near/5 (worker? or visitor? or attendant? or aide or aides or support* or person* or helper? or carer? or caregiver? or care giver? or consultant? or assistant? or staff)):ti,ab,kw
#4	(paraprofessional? or paramedic or paramedics or paramedical worker? or paramedical personnel or allied health personnel or allied health worker? or support worker? or home health aide?):ti,ab,kw
#5	((community or village? or lay) near/3 (health worker? or health care worker? or healthcare worker?)):ti,ab,kw
#6	(doula? or douladural? or barefoot doctor?):ti,ab,kw
#7	{or #1‐#6}
#8	MeSH descriptor: [Cell Phones] this term only
#9	MeSH descriptor: [Smartphone] this term only
#10	MeSH descriptor: [MP3‐Player] this term only
#11	MeSH descriptor: [Computers, Handheld] this term only
#12	((cell* or mobile*) near/1 (phone* or telephone* or technolog* or device*)):ti,ab,kw
#13	(handheld or hand‐held):ti,ab,kw
#14	(smartphone* or smart‐phone* or cellphone* or mobiles):ti,ab,kw
#15	((personal near/1 digital) or (PDA near/3 (device* or assistant*)) or MP3 player* or MP4 player*):ti,ab,kw
#16	(samsung or nokia):ti,ab,kw
#17	(windows near/3 (mobile* or phone*)):ti,ab,kw
#18	android:ti,ab,kw
#19	(ipad* or i‐pad* or ipod* or i‐pod* or iphone* or i‐phone*):ti,ab,kw
#20	(tablet* near/3 (device* or computer*)):ti,ab,kw
#21	MeSH descriptor: [Telemedicine] this term only
#22	MeSH descriptor: [Videoconferencing] this term only
#23	MeSH descriptor: [Webcasts as Topic] this term only
#24	MeSH descriptor: [Text Messaging] this term only
#25	MeSH descriptor: [Telenursing] this term only
#26	(mhealth or m‐health or "mobile health" or ehealth or e‐health or "electronic health"):ti,ab,kw
#27	(telemedicine or tele‐medicine or telehealth or tele‐health or telecare or tele‐care or telenursing or tele‐nursing or telepsychiatry or tele‐psychiatry or telemonitor* or tele‐monitor* or teleconsult* or tele‐consult* or telecounsel* or tele‐counsel* or telecoach* or tele‐coach*):ti,ab,kw
#28	(videoconferenc* or video‐conferenc* or webcast* or web‐cast*):ti,ab,kw
#29	(((text* or short or voice or multimedia or multi‐media or electronic or instant) near/1 messag*) or instant messenger) .ti,ab,kw
#30	(texting or texted or texter* or ((sms or mms) near (service* or messag*)) or interactive voice response* or IVR or voice call* or callback* or voice over internet or VOIP):ti,ab,kw
#31	(Facebook or Twitter or Whatsapp* or Skyp* or YouTube or "You Tube" or Google Hangout*):ti,ab,kw
#32	MeSH descriptor: [Mobile Applications] this term only
#33	"mobile app*":ti,ab,kw
#34	MeSH descriptor: [Social Media] this term only
#35	(social near (media or network*)):ti,ab,kw
#36	MeSH descriptor: [Reminder Systems] this term only
#37	(remind* near/3 (text* or system* or messag*)):ti,ab,kw
#38	MeSH descriptor: [Electronic Mail] this term only
#39	(electronic mail* or email* or e‐mail or webmail):ti,ab,kw
#40	MeSH descriptor: [Medical Informatics] this term only
#41	MeSH descriptor: [Medical Informatics Applications] this term only
#42	MeSH descriptor: [Nursing Informatics] this term only
#43	MeSH descriptor: [Public Health Informatics] this term only
#44	((medical or clinical or health or healthcare or nurs*) near/3 informatics):ti,ab,kw
#45	MeSH descriptor: [Multimedia] this term only
#46	MeSH descriptor: [Hypermedia] this term only
#47	MeSH descriptor: [Blogging] this term only
#48	(multimedia or multi‐media or hypermedia or hyper‐media or blog* or vlog* or weblog* or web‐log*):ti,ab,kw
#49	MeSH descriptor: [Interactive Tutorial] this term only
#50	MeSH descriptor: [Computer‐Assisted Instruction] this term only
#51	((interactive or computer‐assisted) near/1 (tutor* or technolog* or learn* or instruct* or software or communication)):ti,ab,kw
#52	{or #8‐#51}
#53	#7 and #52 Publication Year from 2000 to 2018 in Trials

**Popline – Searched 25 September 2018**

Keyword:(TEXT MESSAGING OR MOBILE DEVICES OR INFORMATION COMMUNICATION TECHNOLOGY OR CELLULAR PHONE) OR All Fields: ((cell OR cellular OR mobile) AND (phone OR phones OR telephone OR telephones OR technology OR technologies OR device OR devices)) OR smartphone OR smartphones OR smart‐phone OR smart‐phones OR cellphone OR cellphones OR mobiles OR mhealth OR m‐health OR "mobile health" OR ehealth OR e‐health OR "electronic health" OR telemedicine OR tele‐medicine OR telehealth OR tele‐health OR telecare OR tele‐care OR telenursing OR tele‐nursing OR telepsychiatry OR tele‐psychiatry OR telemonitor OR telemonitoring OR tele‐monitor OR tele‐monitoring OR teleconsult OR teleconsulting OR tele‐consult OR tele‐consulting OR telecounsel OR telecounseling OR tele‐counsel OR tele‐counseling OR telecoach OR telecoaching OR tele‐coach OR tele‐coaching OR videoconference OR videoconferences OR videoconferencing OR video‐conference OR video‐conferences OR video‐conferencing OR webcast OR webcasts OR webcasting OR web‐cast OR web‐casts OR web‐casting OR ((text OR texts OR texting OR short OR voice OR multimedia OR multi‐media OR electronic OR instant) AND (message OR messages OR messaging)) OR "instant messenger" OR texting OR texted OR texter OR texters OR ((sms OR mms) AND (service OR services OR message OR messages OR messaging)) OR "interactive voice response" OR "interactive voice responses" OR ivr OR "voice call" OR "voice calls" OR callback OR "voice over internet" OR voip OR "mobile app" OR "mobile apps" OR "mobile application" OR "mobile applications" OR "social media" OR ((medical OR clinical OR health OR healthcare OR nurse OR nurses OR nursing) AND informatics)

AND

Keyword: (QUANTITATIVE RESEARCH OR RESEARCH METHODOLOGY OR CLINICAL TRIALS OR CONTROL GROUPS) OR All Fields: (randomised OR randomized OR "randomly allocated" OR "random allocation" OR "controlled trial" OR "control group" OR "control groups" OR trial) (2000‐2018)

**WHO Global Health Library (Regional Indexes only) – 25 September 2018**

(mh:(("cell phones" OR smartphone OR mp3‐player OR "Computers, Handheld" OR telemedicine OR Videoconferencing OR "Text Messaging" OR Telenursing OR "Mobile Applications" OR "Reminder Systems" OR "Electronic Mail" OR "Medical Informatics" OR "Nursing Informatics" OR "Public Health Informatics" OR Multimedia OR Hypermedia OR Blogging OR Telemedicine))) OR (tw:(("cell phone" OR "cell phones" OR "cellular phone" OR "cellular phones" OR "mobile phone" OR "mobile phones" OR "mobile devices" OR "mobile devices" OR smartphone OR smartphones OR smart‐phone OR smart‐phones OR cellphone OR cellphones))) AND (mh:(("Controlled Clinical Trials, Randomized" OR "Controlled Clinical Trials as Topic" OR "Controlled Clinical Trial" OR "Clinical Trial"))) OR (tw:((randomised OR randomized OR "randomly allocated" OR "random allocation" OR "controlled trial" OR "control group" OR "control groups" OR trial))) – 598 hits (2000‐2018)

**ClinicalTrials.gov – 25 September 2018**

Field search: Other Terms: (telemedicine OR telehealth OR telecare OR telenursing OR telepsychiatry OR mhealth OR ehealth) AND ("mobile phone" OR "mobile phones" OR "mobile devices" OR mobiles OR smartphone OR smartphones) | Studies received on or after 01/01/2000 | Studies updated on or before 25/09/2018

**WHO ICTRP – 25 September 2018**

Search 1:

Title: telemedicine OR telehealth OR telecare OR telenursing OR telepsychiatry OR mhealth OR ehealth

AND

Intervention: mobile device OR mobiles OR smartphone OR phone OR cellphone

Search 2:

Title: mobile device OR mobiles OR smartphone OR phone OR cellphone

AND

Intervention: telemedicine OR telehealth OR telecare OR telenursing OR telepsychiatry OR mhealth OR ehealth

## Data and analyses

**Comparison 1:** Mobile technologies used by primary care providers to consult with a hospital‐based specialist compared to usual care: Providers' adherence to recommended practice, guidelines or protocols

Outcome or subgroup title	No. of studies	No. of participants	Statistical method	Effect size
1.1 Providers' adherence to recommended guidelines	1		Other data	No numeric data

**Comparison 2:** Mobile technologies used by primary care providers to consult with a hospital‐based specialist compared to usual care: Time between presentation and management of the health condition

Outcome or subgroup title	No. of studies	No. of participants	Statistical method	Effect size
2.1 Time between presentation and management	4		Other data	No numeric data

**Comparison 3:** Mobile technologies used by primary care providers to consult with a hospital‐based specialist compared to usual care: Healthcare use

Outcome or subgroup title	No. of studies	No. of participants	Statistical method	Effect size
3.1 Healthcare use	9		Other data	No numeric data
3.1.1 Healthcare use	9		Other data	No numeric data
3.2 Referred for clinic follow‐up or clinical examination, 3 to 12 months follow‐up	3		Risk Ratio (M‐H, Random, 95% CI)	Totals not selected
3.2.1 Referred to a dermatology clinic	3		Risk Ratio (M‐H, Random, 95% CI)	Totals not selected
3.3 Referred for clinic follow‐up or clinical examination, 3 to 12 months follow‐up	2		Risk Ratio (M‐H, Random, 95% CI)	Totals not selected

**Comparison 4:** Mobile technologies used by primary care providers to consult with a hospital‐based specialist compared to usual care: Participant's healthcare status and well‐being

Outcome or subgroup title	No. of studies	No. of participants	Statistical method	Effect size
4.1 Health‐related quality of life	2		Other data	No numeric data
4.2 Clinical course	2		Other data	No numeric data

**Comparison 5:** Mobile technologies used by primary care providers to consult with a hospital‐based specialist compared to usual care: Acceptability or satisfaction

Outcome or subgroup title	No. of studies	No. of participants	Statistical method	Effect size
5.1 Healthcare provider satisfaction with the intervention	3		Other data	No numeric data
5.2 Participant satisfaction with care	4		Other data	No numeric data

**Comparison 6:** Mobile technologies used by primary care providers to consult with a hospital‐based specialist compared to usual care: Costs

Outcome or subgroup title	No. of studies	No. of participants	Statistical method	Effect size
6.1 Costs	6		Other data	No numeric data

**Comparison 7:** Mobile technologies used by primary care providers to consult with a hospital‐based specialist compared to usual care: Technical difficulties

Outcome or subgroup title	No. of studies	No. of participants	Statistical method	Effect size
7.1 Technical difficulties	4		Other data	No numeric data
7.1.1 Quality of the data transmitted	4		Other data	No numeric data

**Comparison 8:** Mobile technologies for use in the emergency department compared to usual care: Time between presentation and management of the health condition

Outcome or subgroup title	No. of studies	No. of participants	Statistical method	Effect size
8.1 Time between presentation and management	1		Other data	No numeric data

**Comparison 9:** Mobile technologies for use in the emergency department compared to usual care: Healthcare use

Outcome or subgroup title	No. of studies	No. of participants	Statistical method	Effect size
9.1 Healthcare use	1		Other data	No numeric data

**Comparison 10:** Mobile technologies for use in the emergency department compared to usual care: Technical difficulties

Outcome or subgroup title	No. of studies	No. of participants	Statistical method	Effect size
10.1 Technical difficulties	1		Other data	No numeric data
10.1.1 Quality of the data transmitted	1		Other data	No numeric data

**Comparison 11:** Mobile technologies used by community health workers or home‐care workers compared to usual care: Healthcare use

Outcome or subgroup title	No. of studies	No. of participants	Statistical method	Effect size
11.1 Healthcare use	2		Other data	No numeric data

**Comparison 12:** Mobile technologies used by community health workers or home‐care workers compared to usual care: Participant's healthcare status and well‐being

Outcome or subgroup title	No. of studies	No. of participants	Statistical method	Effect size
12.1 Participant healthcare status and well‐being	3		Other data	No numeric data

**Comparison 13:** Mobile technologies used by community health workers or home‐care workers compared to usual care: Acceptability or satisfaction

Outcome or subgroup title	No. of studies	No. of participants	Statistical method	Effect size
13.1 Participant satisfaction with care	2		Other data	No numeric data

**Comparison 14:** Mobile technologies used by community health workers or home‐care workers compared to usual care: Costs

Outcome or subgroup title	No. of studies	No. of participants	Statistical method	Effect size
14.1 Costs	1		Other data	No numeric data

**Comparison 15:** Mobile technologies used by community health workers or home‐care workers compared to usual care: Technical difficulties

Outcome or subgroup title	No. of studies	No. of participants	Statistical method	Effect size
15.1 Technical difficulties	2		Other data	No numeric data
15.1.1 Quality of the data transmitted	1		Other data	No numeric data
15.1.2 Technical difficulties reported by the healthcare professionals	1		Other data	No numeric data

## Characteristics of studies

### Characteristics of included studies [ordered by study ID]

**Table:** Armstrong 2018

***Study characteristics***		
Methods	**Study design:** Randomised trial (parallel assignment) **Unit of allocation:** Participant (GP)	
Participants	**Providers** **Number:** 296 participants randomised (same number ITT); number of professionals not described **Type:** GPs consulting with dermatologists in secondary care **Other relevant characteristics:** none reported **Participants** **Number:** Eligible: I: 148; C: 148 Analysed: same number (ITT) **Mean age (SD):** 49 years (14) **Gender (% female):** 50% **Inclusion criteria:** Adults diagnosed with plaque psoriasis, access to Internet and digital camera or mobile phone with camera **Exclusion criteria:** No diagnosis of plaque psoriasis, living outside the designated catchment area **Other relevant characteristics:** Mainly white (63%), with college education or more (88%), and working full‐time (47%). Baseline scores for severity of the condition were lower than anticipated, as several participants were receiving other therapies **Location and study setting:** USA, 3 regions **Recruitment method:** Participants recruited from practice‐based research networks, federally‐qualified health centres, and university‐based clinics, national groups and general public **Duration:** Number of sessions varied by participant, 12‐months follow‐up. Study ran between 2 February 2015 and 18 August 2017 **Withdrawals:** 6% of randomised participants were lost to follow‐up and 4% withdrew	
Interventions	**Intervention components:** Online, collaborative connected‐health model between participant, PCP and dermatologist. PCP could communicate with the dermatologist using the consultation function, sending digital photographs and clinical history for discussion. The dermatologist would assess the data and reply to the PCP within 2 business days, recommending treatment as well as educational materials for the participant. With the PCP permission, the dermatologist could also contact the participant directly. The PCP could also request for the dermatologist to become the main HCP. All communication was done through a secure web‐based platform. Participants were paid for participating in the study, through gift cards **Comparison:** Usual care: in‐person care as needed, frequency established by participants and their providers **Technical equipment used:** Secure safety policy–compliant web‐based connected‐health platform; mobile phone or digital camera for collecting images **Fidelity assessment:** Not reported; protocol states that protocol deviations will be noted, but not how they will be assessed	
Outcomes	**Main outcome:** Self‐reported psoriasis severity **Other outcomes:** Quality of life, access to care; depression; disease severity **Time points reported:** Baseline, 3‐, 6‐, 9‐, 12‐months	
Notes	**Funding:** Patient‐Centered Outcomes Research Institute Award (IHS‐071502‐IC) **Ethical approval:** Approved by university institutional review boards. Trial registry NCT02358135 **Conflicts of interest:** "Dr Armstrong reported serving as an investigator, consultant, advisor, and/or speaker for AbbVie, Janssen, Lilly, Novartis, Sanofi, Regeneron, Leo, Science 37, Modmed, Pfizer, Ortho Dermatologics, and Modernizing Medicine. Dr Gelfand reported serving as a consultant for and receiving honoraria from BMS, Coherus (DSMB), Dermira, GSK, Janssen Biologics, Menlo Therapeutics, Novartis Corp, Regeneron, Dr Reddy’s Laboratories, Sanofi, and Pfizer Inc; receiving research grants (to the Trustees of the University of Pennsylvania) from AbbVie, Janssen, Novartis Corp, Regeneron, Sanofi, Celgene, Ortho Dermatologics, and Pfizer Inc; receiving payment for continuing medical education work related to psoriasis that was supported indirectly by Lilly, Ortho Dermatologics, and AbbVie; and being a co–patent holder of resiquimod for treatment of cutaneous T‐cell lymphoma. Dr Wong reported being an employee of DirectDerm. No other disclosures were reported."	
***Risk of bias***		
**Bias**	**Authors' judgement**	**Support for judgement**
Random sequence generation (selection bias)	Low risk	Comment: Computer‐generated random block sizes (p.3)
Allocation concealment (selection bias)	Low risk	Comment: Independent statistician (p.4)
Baseline outcome measurements similar (selection bias)	Low risk	Comment: Baseline data provided for main outcome and similar between groups (Table 1)
Baseline characteristics similar (selection bias)	Low risk	Comment: Baseline characteristics provided and similar between groups (Table 1)
Blinding of participants and personnel (performance bias) All outcomes	High risk	Comment: Not possible to blind participants or personnel
Blinding of objective outcome assessment (detection bias)	Low risk	Comment: Data analyst blinded
Blinding of subjective outcome assessment (detection bias)	High risk	Comment: Patient‐reported outcome measures, unblinded participants and personnel
Protection against contamination	High risk	Comment: Patients were randomised to the groups
Incomplete outcome data (attrition bias) All outcomes	Low risk	Comment: Intention‐to‐treat analysis
Selective reporting (reporting bias)	High risk	Comment: Not all outcomes specified in the protocol reported in publications (distance travel, wait time)
Other bias	Low risk	Comment: No other apparent source of bias

**Table:** Azogil‐López 2019

***Study characteristics***		
Methods	**Study design:** Randomised trial (parallel assignment) **Unit of allocation:** Participant (GP)	
Participants	**Providers** **Number:** 58 GPs randomised (31 analysed), number of hospital‐based physicians not reported **Type:** GPs consulting with physicians in secondary care **Other relevant characteristics:** GPs' median age was approximately 57 years, on average 34 km away from the hospital **Participants** **Number:** Eligible: I: 92; C: 164 Analysed: I: 72; C: 101 **Median age:** I: 56 years; C: 55 years **Gender (% female):** I: 59%; C: 60% **Inclusion criteria:** Adults who consulted with the GP and required a referral to secondary care **Exclusion criteria:** People who required or preferred an in‐person appointment **Other relevant characteristics:** Not reported **Location and study setting:** Spain, 6 primary care practices **Recruitment method:** Not reported **Duration:** Single session; 3‐months follow‐up. Study ran between March and December 2016 **Withdrawals:** 38% (N = 19) of eligible GPs were excluded from analysis (IG: 6 GPs excluded as they did not request phone consultations with physicians; CG: 13 GPs excluded as they did not collect data for at least 50% of their eligible participants); of those receiving care by GPs allocated to the IG, 50% were excluded from analysis as they were given in‐person appointments (either because they required or preferred it)	
Interventions	**Intervention components:** If during an initial appointment the GP considered a referral for a speciality appointment was needed, the GP would request an eConsult with the specialist, which would take place at the primary care practice. The GP would call the consultant at a convenient time, while the participant was still in the room. The 2 healthcare professionals would agree on the treatment, whether further investigations were required, and book follow‐up appointments **Comparison:** Usual care ‐ participant was given a referral for an in‐person appointment at secondary care **Technical equipment used:** Hands‐free telephone **Fidelity assessment:** Not reported	
Outcomes	**Main outcomes:** Waiting days between the GP referring the participant for an appointment and the appointment being provided; number of avoided/avoidable face‐to‐face referrals; waiting days for the resolution of the process **Time points reported:** Post‐intervention (3‐months follow‐up)	
Notes	**Funding:** Andalusian Society of Family and Community Medicine ‐SAMFyC‐ (Record ref. 157/18) **Ethical approval:** Regional research ethics committee. Trial registry ACTRN12617001536358 **Conflicts of interest:** None known	
***Risk of bias***		
**Bias**	**Authors' judgement**	**Support for judgement**
Random sequence generation (selection bias)	Low risk	Comment: Random‐number table (p. 3)
Allocation concealment (selection bias)	Low risk	Comment: Unit of allocation was by primary care practices, and allocation was performed on all units at the start of the study (p.3)
Baseline outcome measurements similar (selection bias)	Unclear risk	Comment: Not enough information provided
Baseline characteristics similar (selection bias)	High risk	Comment: Baseline differences between groups about participants distance to hospital and geographical living area (table 2)
Blinding of participants and personnel (performance bias) All outcomes	High risk	Comment: Not possible to blind participants or personnel
Blinding of objective outcome assessment (detection bias)	Low risk	Comment: Automatically extracted from the EMR (p.4)
Protection against contamination	Low risk	Comment: Allocation by healthcare providers
Incomplete outcome data (attrition bias) All outcomes	High risk	Comment: High attrition rates (almost 40% randomised GPs excluded from analysis; p.4)
Selective reporting (reporting bias)	Low risk	Comment: All outcomes listed in Methods reported in Results
Other bias	Low risk	Comment: No other apparent source of bias

**Table:** Byamba 2015

***Study characteristics***		
Methods	**Study design:** Cluster‐randomised trial (parallel assignment) **Unit of allocation:** Cluster (clinics)	
Participants	**Providers** **Number:** 20 GPs, number of hospital‐based physicians not reported **Type:** GPs consulting with physicians in secondary care **Other relevant characteristics:** Not reported **Participants** **Number:** Eligible: I: 221; C: 229 Analysed: same number **Median age:** Not reported **Gender (% female):** Not reported **Inclusion criteria:** Adults who consulted with the GP and required a referral to secondary care for skin lesions and problems **Exclusion criteria:** Not reported **Other relevant characteristics:** Not reported **Location and study setting:** Mongolia, 20 rural health clinics in 1 of the least densely‐population countries in the world. **Recruitment method:** Not reported **Duration:** 5‐month follow‐up. Study ran between September 2013 and January 2014 **Withdrawals:** Not reported	
Interventions	**Intervention components:** A primary care provider in a rural health clinic used a smartphone camera to collect images and clinical history of participants with skin lesions and problems. The PCP attended a 2‐day training session to learn how to take images and use the medical record system and software on mobile phones. The information was sent along with a teleconsultation request using the electronic medical record. The dermatologist reviewed the information and sent feedback within 24 hours. **Comparison:** Usual care ‐ GPs referred participants to district hospitals or the National Dermatology Centre **Technical equipment used:** Android‐based system **Fidelity assessment:** GPs from clinics allocated to the intervention attended a 2‐day training session on how to take pictures using the devices provided and how to operate the electronic medical record	
Outcomes	**Main outcomes:** Tertiary care referrals; costs **Time points reported:** Post‐intervention (5‐months follow‐up)	
Notes	**Funding:** National science Council Project no. NSC 101‐2923‐E‐038 ‐001 ‐MY2, Ministry of Health and Welfare (MOHW), Taiwan, under grant MOHW103‐TD‐B‐111‐01 and Taipei Medical University under grant 101TMUSHH‐21 **Ethical approval:** Not reported **Conflicts of interest:** None known	
***Risk of bias***		
**Bias**	**Authors' judgement**	**Support for judgement**
Random sequence generation (selection bias)	Low risk	Comment: Computer‐generated sequence
Allocation concealment (selection bias)	Low risk	Comment: Cluster randomisation, all done at the start of the study
Baseline outcome measurements similar (selection bias)	Unclear risk	Comment: Not enough information provided
Baseline characteristics similar (selection bias)	Unclear risk	Comment: Not reported
Blinding of participants and personnel (performance bias) All outcomes	Unclear risk	Comment: Not reported
Blinding of objective outcome assessment (detection bias)	Unclear risk	Comment: Not reported how it was collected
Protection against contamination	Low risk	Comment: Allocation was by practice, unlikely that the control group received the intervention
Incomplete outcome data (attrition bias) All outcomes	Low risk	Comment: There was no attrition
Selective reporting (reporting bias)	Low risk	Comment: All outcomes mentioned in Methods are presented in Results
Other bias	Low risk	Comment: No other apparent source of bias

**Table:** Chang 2011

***Study characteristics***		
Methods	**Study design:** Cluster‐randomised trial (parallel) **Unit of allocation:** Cluster (10 clinics; I: 4, C: 6)	
Participants	**Providers** **Number:** I: 13, C: 16 **Type:** Community‐based peer health workers consulting with clinic staff **Other relevant characteristics:** None reported **Participants** **Number:** Randomised: 970 (I: 446, C: 524), Analysed: ITT analysis **Median age (range):** 35 years (15‐76) **Gender (% female):** 66% **Inclusion criteria:** Adults attending eligible clinics who were receiving or started receiving ART **Exclusion criteria:** None reported **Other relevant characteristics:** None reported **Location and study setting:** Uganda, 10 clinic sites **Recruitment method:** Not applicable; participants were not informed about the study as PHWs were performing routine care functions **Duration:** Intervention lasted 26 weeks, median follow‐up time was 103 weeks (97 ‐ 111 weeks), study was conducted between May 2006 and July 2008 **Withdrawals:** 11% and 12% of participants allocated to Intervention and Control were lost to follow‐up; main reason was death (8.3% and 10.1% of all participants lost, respectively)	
Interventions	**Intervention components:** mHealth intervention: Periodic home visits, supported by mobile phone. PHWs participated in a 1‐day residential training and were given a mobile phone and an hour‐long field‐based practicum. After each home visit the PHW would message data on adherence and other clinical information to a centralised database, which was staffed by clinic staff. Once a message had been received, clinic staff could provide care instructions, send a higher‐level care provider, or arrange for the participant to be taken to a healthcare facility. PHWs could also call a hotline if they had questions **Comparison:** No mobile phone, additional training or access to the hotline. All PHWs had previously been enrolled in a study of ART provision, where they received a 2‐day residential training on HIV‐related topics, as well as adherence counselling, patient confidentiality and filling out home visit forms. The main goal of the home visits was to evaluate and encourage adherence to ART therapy **Technical equipment used:** mobile phones (no further details provided) **Fidelity assessment:** PHWs were supervised by a member of staff (part‐time worker)	
Outcomes	**Main outcomes:** Participants’ cumulative risk of virologic failure **Other outcomes:** Participant adherence; virologic failure at 24 and 48 weeks of ART; lost to follow‐up; mortality; qualitative evaluation (interviews, themes included impact of the intervention, confidentiality concerns and challenges with phones); cost analyses (intervention arm only) **Time points reported:** Baseline, post‐intervention (median follow‐up was 103 weeks post‐baseline)	
Notes	**Funding:** Doris Duke Charitable Foundation, The Division of Intramural Research, The National Institute for Allergy and Infectious Diseases, National Institutes of Health, and a National Institutes of Health Training Grant and Career Development Grant **Ethical approval:** Institutional review boards at the Uganda Virus Research Institute’s Safety and Ethics Committee, the Uganda National Council for Science and Technology, and Johns Hopkins University **Conflicts of interest:** Not reported	
***Risk of bias***		
**Bias**	**Authors' judgement**	**Support for judgement**
Random sequence generation (selection bias)	Unclear risk	Comment: Inadequate information reported, no description of sequence generation ‐ Quote: "The 10 sites (...) randomised 2:3 to PHWs receiving a mHealth support intervention or not" (p.3)
Allocation concealment (selection bias)	Low risk	Comment: Cluster randomisation, all clinics done at the start of the study
Baseline outcome measurements similar (selection bias)	Low risk	Quote: "[K]ey predictors of clinical outcomes appeared well balanced between arms." (p.4)
Baseline characteristics similar (selection bias)	Low risk	Comment: Reported and similar between groups (Table 1)
Blinding of participants and personnel (performance bias) All outcomes	High risk	Comment: Not possible to blind participants or personnel
Blinding of objective outcome assessment (detection bias)	Unclear risk	Comment: No information provided on how objective outcomes were collected
Protection against contamination	Low risk	Comment: Cluster‐randomised trial with participants/peer health workers with mobile phone intervention access in separate districts
Incomplete outcome data (attrition bias) All outcomes	High risk	Comment: High attrition rate
Selective reporting (reporting bias)	High risk	Comment: Outcomes reported in different publications
Other bias	Low risk	Comment: No other apparent source of bias

**Table:** Davis 2003

***Study characteristics***		
Methods	**Study design:** Randomised trial (parallel) **Unit of allocation:** Participant	
Participants	**Providers** **Number:** Two **Type:** Primary care provider at the rural primary practice consulting with ophthalmologist in the university setting **Other relevant characteristics:** None reported **Participants** **Number:** Randomised: 59 (I: 30, C: 29), Analysed: same number **Mean age (SD):** Not reported **Gender (% female):** Not reported **Inclusion criteria:** Adults with diabetes diagnosed by a physician **Exclusion criteria:** Not reported **Other relevant characteristics:** Mainly African‐Americans **Location and study setting:** USA, 1 rural primary practice and 1 urban university hospital **Recruitment method:** Not reported **Duration:** Not reported **Withdrawals:** Not reported; all participants analysed	
Interventions	**Intervention components:** A primary care provider in a rural primary care practice used a nonmydriatic retinal camera and video‐conferencing to send real‐time images to an ophthalmologist located in an urban university setting. The ophthalmologist assessed the retinal photograph and communicated with the participant and the primary care professional **Comparison:** Usual care ‐ participants were reminded to schedule examinations with their usual eye‐care provider **Technical equipment used:** nonmydriatic retinal camera (Topcon with IMAGEnet software) and video‐conferencing (no further details provided) **Fidelity assessment:** Not reported	
Outcomes	**Main outcomes:** Frequency of eye examinations **Time points reported:** Post‐intervention	
Notes	**Funding:** Not reported **Ethical approval:** Not reported **Conflicts of interest:** Not reported **Notes:** short reports (letter and abstract), limited information	
***Risk of bias***		
**Bias**	**Authors' judgement**	**Support for judgement**
Random sequence generation (selection bias)	Unclear risk	Comment: Not enough information provided
Allocation concealment (selection bias)	Unclear risk	Comment: Not enough information provided
Baseline outcome measurements similar (selection bias)	Unclear risk	Comment: Not enough information provided
Baseline characteristics similar (selection bias)	Unclear risk	Comment: Not enough information provided
Blinding of participants and personnel (performance bias) All outcomes	Unclear risk	Comment: Not enough information provided
Blinding of objective outcome assessment (detection bias)	Unclear risk	Comment: Not enough information provided
Blinding of subjective outcome assessment (detection bias)	Unclear risk	Comment: Not enough information provided
Protection against contamination	Unclear risk	Comment: Not enough information provided
Incomplete outcome data (attrition bias) All outcomes	Unclear risk	Comment: Not enough information provided
Selective reporting (reporting bias)	Unclear risk	Comment: Not enough information provided
Other bias	Unclear risk	Comment: Not enough information provided

**Table:** Eminović 2009

***Study characteristics***		
Methods	**Study design:** Cluster‐randomised trial (parallel) **Unit of allocation:** Cluster (36 GP practices; I: 19; C: 17)	
Participants	**Providers** **Number:** GPs: I: 59, C: 51; Dermatologists: 5 **Type:** GPs consulting with dermatologists **Other relevant characteristics:** GPs: I: 29% female, C: 35% female **Participants** **Number:** Randomised: 631 (I: 327, C: 304), Analysed: 605 (I: 312, C: 293) **Mean age (SD):** I: 42 years (23); C: 44 years (20) **Gender (% female):** I: 56%; C: 64% **Inclusion criteria:** Practices were required to have facilities to send digital images over the Internet; participants were eligible if they were referred to a dermatologist by their GP **Exclusion criteria:** GPs who already used teledermatology; patients were excluded if they required an urgent dermatology appointment **Other relevant characteristics:** None reported **Location and study setting:** The Netherlands, 36 primary care practices **Recruitment method:** Dermatologists working in eligible areas were invited to participate; GPs working in practices that referred participants to those dermatologists were then invited to participate **Duration:** Intervention was 1 teleconsultation, with 1 month follow‐up; study conducted between February 2004 and January 2006 **Withdrawals:** 5% (I) and 7% (C) of participants randomised were lost to follow‐up, main reasons were problems with data entry and participants visiting another dermatologist; for 39% of participants, information on the main outcome was missing, mainly because GPs did not complete study forms	
Interventions	**Intervention components:** GPs allocated to the intervention group received detailed instructions on how to take digital images and use the web‐based form. GPs took 4 digital images of the skin problems and completed a structured form (which included questions about duration and location of the skin lesion) on a secure website; the form was sent to the dermatologist along with the main reason for referral (diagnosis, advice, reassurance). At this stage GPs could also refer the participant to another dermatologist. Within 48 hours the dermatologist assessed the images and replied to the GP using the same system, providing advice and whether further investigations or urgent referrals were required. After a month the dermatologist saw the participant in person, regardless of the outcome of the online consultation **Comparison:** Usual care ‐ participants saw a dermatologist according to the usual procedures, usually by being referred by the GP who would give the participant a letter to take to the clinic **Technical equipment used:** digital cameras (Kodak EasyShare CX6230 2.0 megapixel) and a secure teledermatology website **Fidelity assessment:** Not reported	
Outcomes	**Main outcomes:** Proportion of and reasons for preventable consultations **Other outcomes:** Participant satisfaction in general and about interpersonal aspects of the consultation; costs **Time points reported:** 1 month follow‐up	
Notes	**Funding:** Senter Novem (Agency of the Dutch Ministry of Economic Affairs) and ZonMw (Dutch Organization for Health Research and Development). KSYOS Health Management Research provided the digital cameras and teleconsultation software **Ethical approval:** The ethics committee deemed this study to be exempt from review because the research did not interfere with usual care. Trial registration ISRCTN57478950 **Conflicts of interest:** None known	
***Risk of bias***		
**Bias**	**Authors' judgement**	**Support for judgement**
Random sequence generation (selection bias)	Low risk	Quote: "Using dedicated randomisation software, practices were assigned to teledermatologic consultation or standard care." (p.559)
Allocation concealment (selection bias)	Low risk	Quote: "A special allocation concealment procedure (...) was followed to ensure that no allocation bias could occur." (p.559)
Baseline outcome measurements similar (selection bias)	Low risk	Comment: Diagnostic categories well‐balanced between groups (Table 4)
Baseline characteristics similar (selection bias)	Low risk	Comment: Baseline characteristics reported and similar between groups (Table 2)
Blinding of participants and personnel (performance bias) All outcomes	High risk	Comment: Providers could not be blinded, no information on blinding of participants
Blinding of objective outcome assessment (detection bias)	Unclear risk	Comment: Not enough information provided
Blinding of subjective outcome assessment (detection bias)	High risk	Comment: Subjective assessment of whether in‐person consultations could have been prevented done by consulting dermatologist, who knew which participants they had seen
Protection against contamination	Unclear risk	Comment: Treatment and diagnosis of GPs in intervention clinics may have been influenced cumulatively by consultations and contact with dermatologists
Incomplete outcome data (attrition bias) All outcomes	High risk	Comment: High rate of missing data for the primary outcome
Selective reporting (reporting bias)	High risk	Comment: Trial registration states outcomes that were not reported (diagnostic accuracy, delay in treatment, learning effect GPs)
Other bias	Unclear risk	Comment: There were considerable problems following up participants and several methods have been reported to gather outcome data and analyse in different ways

**Table:** Gulacti 2017

***Study characteristics***		
Methods	**Study design:** Randomised trial (parallel) **Unit of allocation:** Participants	
Participants	**Providers** **Number:** Not provided **Type:** Emergency physicians consulting with specialist physicians **Other relevant characteristics:** Not provided **Participants** **Number:** Randomised: 345 (IG: 173, CG: 172), Analysed: same number **Mean age (SD):** 48.5 years (22.1) **Gender (% female):** 33% **Inclusion criteria:** Participants: adults attending the emergency department; Physicians: owned a smartphone and were familiarised with secure messaging applications **Exclusion criteria:** Not reported **Other relevant characteristics:** Not reported **Location and study setting:** Turkey, 1 hospital **Recruitment method:** Not reported **Duration:** Intervention was consultation request using 2 different methods; study was conducted between November 2015 and February 2016 **Withdrawals:** No withdrawals or losses to follow‐up	
Interventions	**Intervention components:** Emergency physician requested consultation with a specialist physician using a secure messaging service (Whatsapp). Any additional medical information (e.g. blood pressure, x‐rays, ultrasounds, photographs) was sent through the same service **Comparison:** Usual care; consultations were requested by telephone, with any additional medical information (e.g. blood pressure, sensory‐motor findings, Glasgow Coma Score) sent verbally **Technical equipment used:** Smartphone with Whatsapp (owned by the healthcare professionals) **Fidelity assessment:** Not reported	
Outcomes	**Main outcomes:** Difference between groups for emergency department length of stay **Other outcomes:** Difference between groups in consult time (time when consultation was requested minus time when a bed was requested or discharge time); termination of consultation between groups **Time points reported:** Baseline, post‐intervention	
Notes	**Funding:** Not reported **Ethical approval:** Medical Ethics Committee. Trial registry NCT02586779 **Conflicts of interest:** None known	
***Risk of bias***		
**Bias**	**Authors' judgement**	**Support for judgement**
Random sequence generation (selection bias)	Low risk	Comment: Computer programme (p.744)
Allocation concealment (selection bias)	Unclear risk	Comment: Not enough information
Baseline outcome measurements similar (selection bias)	Unclear risk	Comment: Not enough information provided
Baseline characteristics similar (selection bias)	Low risk	Comment: Baseline characteristics provided and similar between groups (Table 1)
Blinding of participants and personnel (performance bias) All outcomes	High risk	Comment: Not possible to blind participants or healthcare professionals, even though consulting physicians were blinded to the purpose of the study (p.744)
Blinding of objective outcome assessment (detection bias)	Low risk	Comment: Data collector was blinded (p.744)
Protection against contamination	Unclear risk	Comment: Healthcare professionals within the same hospital randomised and it is not clear whether communication occurred between them
Incomplete outcome data (attrition bias) All outcomes	Low risk	Comment: No attrition
Selective reporting (reporting bias)	High risk	Comment: Outcomes stated in protocol are different from outcomes reported
Other bias	Low risk	Comment: No other apparent source of bias

**Table:** Iversen 2018

***Study characteristics***		
Methods	**Study design:** Cluster‐randomised trial (parallel) **Unit of allocation:** Cluster (42 sites, 21 each arm)	
Participants	**Providers** **Number:** Not reported **Type:** Community nurses consulting with diabetes specialist nurses and podiatrists **Other relevant characteristics:** Not reported **Participants** **Number:** Randomised: 182 (I: 94, C: 88), Analysed: same number **Mean age (SD):** I: 67.2 years (16.7); C: 65.5 years (16.5) **Gender (% female):** I: 75%; C: 74% **Inclusion criteria:** Adults aged ≥ 20 years with new diabetes‐related foot ulcers **Exclusion criteria:** Repeated ulcer treated in the past 6 months, mental illness, life expectancy < 1 year **Other relevant characteristics:** Diagnosed with diabetes for on average 20 years **Location and study setting:** Norway, 2 hospitals **Recruitment method:** Eligible patients attending 1 of 2 hospitals were invited to participate **Duration:** Intervention length could vary according the clinical needs, participants seen every 6 weeks, maximum follow‐up time as 12 months; not study conducted between September 2012 and June 2016 **Withdrawals:** For objective measures, all participants were followed‐up and included in the analysis; for subjective measures attrition rates were 29% for the IG and 35% for the CG	
Interventions	**Intervention components:** Telemedicine application composed by a mobile phone and an interactive web‐based ulcer record. Consultations happened every 6 weeks and included a written assessment and images taken using the mobile phone, which were then sent through the web to the specialist nurse or the podiatrist, who provided feedback. Any doubts could be further discussed. All staff received training in the use of the web‐based system, as well as in‐person access to hospital clinics to improve their practical skills **Comparison:** Usual care ‐ provided by outpatient clinic, usually scheduled every second week **Technical equipment used:** Smartphone (no further description) **Fidelity assessment:** Functionality was assessed yearly and minor adjustments introduced (protocol); all personnel received training and were following standardised guidelines for treatment of diabetes‐related foot ulcers	
Outcomes	**Main outcomes:** Ulcer healing time **Other outcomes:** Amputation; mortality; consultations; participant satisfaction; participant and healthcare professionals' experiences (qualitative) **Time points reported:** Baseline, post‐intervention (12 months post‐baseline)	
Notes	**Funding:** Norwegian Directorate of Health and Innovation Norway, Western Norway Regional Health Authority, Norwegian Diabetes Association, Western Norway University of Applied Sciences, Norwegian Research Council **Ethical approval:** Western Norway Regional Committee for Medical and Health Research Ethics. Trial registry: NCT01710774 **Conflicts of interest:** None known	
***Risk of bias***		
**Bias**	**Authors' judgement**	**Support for judgement**
Random sequence generation (selection bias)	Low risk	Comment: Randomisation using computer programme (p.97)
Allocation concealment (selection bias)	Low risk	Comment: Done by a person independent of the study (p.97)
Baseline outcome measurements similar (selection bias)	Unclear risk	Comment: Higher proportion of participants in the intervention group had ulcers in the toe area (60.6%) compared with control (38.6%) (p.99)
Baseline characteristics similar (selection bias)	Unclear risk	Comment: Higher proportion of participants in the intervention group had type II diabetes (86.2%) compared with control (71.6%) (p.99)
Blinding of participants and personnel (performance bias) All outcomes	High risk	Comment: Not possible to blind participants or healthcare professionals
Blinding of objective outcome assessment (detection bias)	Low risk	Comment: Electronic records (protocol)
Blinding of subjective outcome assessment (detection bias)	High risk	Comment: Participants and healthcare professionals not blinded, self‐reported
Protection against contamination	Unclear risk	Comment: Not enough information provided
Incomplete outcome data (attrition bias) All outcomes	Low risk	Comment: No attrition for objective outcomes; high attrition for the subjective outcome, intention‐to‐treat analysis
Selective reporting (reporting bias)	High risk	Comment: Several outcomes mentioned in protocol and not reported (e.g. quality of life, depression, anxiety)
Other bias	Low risk	Comment: No other apparent source of bias

**Table:** Liddy 2019a

***Study characteristics***		
Methods	**Study design:** Randomised trial (parallel) **Unit of allocation:** Participants (HCP)	
Participants	**Providers** **Number:** Randomised: IG: 57; CG: 56 **Type:** Primary care practitioners consulting with specialist physicians **Other relevant characteristics:** PCP practicing in Ontario and not currently using eConsult. Mostly male (65%), 20 years since graduation **Participants** **Number:** Specific number of participants not reported; each PCP allocated to IG saw on average 724 participants during the pre‐intervention period (range 11 to 1692), whereas those in CG saw 828 participants during the same period (range 93 to 1971) **Mean age (SD):** Not reported **Gender (% female):** Not reported **Inclusion criteria:** Not reported **Exclusion criteria:** Not reported **Other relevant characteristics:** Not reported **Location and study setting:** Ontario, Canada **Recruitment method:** Eligible PCPs were sent an information pack and invited to participate by a third party; those interested could get directly in touch with the research team **Duration:** Trial conducted between 31 January 2014 and 26 September 2014, 12 months follow‐up from baseline **Withdrawals:** Approximately 12% of PCPs were not analysed at baseline and follow‐up due to lack of referral data	
Interventions	**Intervention components:** Champlain BASE™ eConsult service, a web‐based application that the PCP used to submit participant‐specific clinical questions to specialists. The specialists responded within 7 days, with recommendations, further questions, or recommendation for a face‐to‐face referral. Specialists received financial incentives for each eConsult they undertook **Comparison:** Usual care ‐ standard referral practices **Technical equipment used:** The application could be accessed through smartphone, laptop or desktop; most users accessed it through their smartphones **Fidelity assessment:** Participant PCPs underwent an orientation session and brief training, without which they could not access the system	
Outcomes	**Main outcomes:** Specialist referral rate per 100 participants seen to all medical specialties available through eConsult service **Other outcomes:** Referral rate to all medical specialties **Time points reported:** Baseline, follow‐up	
Notes	**Funding:** Ontario Ministry of Health and Long‐Term Care (MOHLTC) and Health Services Research Fund, Ministry Grant #06547, Province of Ontario, Primary Health Care Program (INSPRE‐PHC). **Ethical approval:** Hospital Research Ethics Board and the Institutional Review Board. Trial registry NCT02053467 **Conflicts of interest:** None known	
***Risk of bias***		
**Bias**	**Authors' judgement**	**Support for judgement**
Random sequence generation (selection bias)	Low risk	Comment: Computer‐generated random list of numbers ("Randomization")
Allocation concealment (selection bias)	Low risk	Comment: Done by an independent research staff member, using opaque sealed envelopes ("Randomization")
Baseline outcome measurements similar (selection bias)	Low risk	Comment: Baseline outcome measurements provided and similar between groups, although IG had slightly lower referral rates at baseline (Table 2, Table 3)
Baseline characteristics similar (selection bias)	Low risk	Comment: Baseline characteristics provided (Table 1); differences between groups for model of practice and practice size, practice model and location adjusted for in the analysis
Blinding of participants and personnel (performance bias) All outcomes	High risk	Comment: Not possible due to the nature of the intervention
Blinding of objective outcome assessment (detection bias)	Low risk	Comment: Automatically extracted from electronic medical records ("Data sources")
Protection against contamination	Unclear risk	Comment: PCPs were randomised to intervention and control groups; unclear whether they could be located in the same practice
Incomplete outcome data (attrition bias) All outcomes	High risk	Comment: Authors note that for missing outcomes, due to limitations related to the use of administrative health databases, they did not end up with 50 providers per arm, resulting in an underpowered trial
Selective reporting (reporting bias)	Low risk	Comment: Outcomes reported as per protocol
Other bias	Low risk	Comment: No other apparent source of bias

**Table:** Mansberger 2015

***Study characteristics***		
Methods	**Study design:** Randomised trial (parallel) **Unit of allocation:** Participant	
Participants	**Providers** **Number:** Primary care professionals not reported, 2 experienced investigators **Type:** Primary care professionals consulting with experienced investigators based at an eye institute **Other relevant characteristics:** **Participants** **Number:** Randomised: 567 (I: 296, C: 271), Analysed (12 months follow‐up): same number **Mean age (SD):** I: 50.2 years (12.3); C: 51.7 years (11.3) **Gender (% female):** I: 52%; C: 51% **Inclusion criteria:** Adults diagnosed with diabetes who were scheduled to visit the primary care provider **Exclusion criteria:** Cognitive impairment **Other relevant characteristics:** On average, diagnosed with diabetes for 10 years. The overall prevalence of diabetic retinopathy (21.5%) was lower than the national average (28.5%) **Location and study setting:** USA, 2 primary care clinics **Recruitment method:** Research assistants called potentially eligible patients and invited them to participate **Duration:** Follow‐up lasted 48 months; not reported when study was conducted **Withdrawals:** 100% response rate at 12 months follow‐up, approximately 76% response rate at 48 months follow‐up	
Interventions	**Intervention components:** Telemedicine ‐ digital images were captured with a non‐mydriatic camera by clinic technicians and sent to a specialist for review and report generation. Technicians performing imaging attended a 3‐day training session to learn how to take images and ongoing feedback as needed. Communication was done through private encrypted software, which transferred images and participant data to a secure database. Experienced investigators would receive an alert once the images were available, and grade them based on international standardised criteria, completing online reports that were automatically sent to clinic staff. Participants were also encouraged to see an eye care provider early as the camera‐based exam is not considered to be a replacement for a comprehensive eye exam. Participants received monetary incentive to complete follow‐up questionnaire **Comparison:** Usual care ‐ during their primary care visit, participants were encouraged to see an eye care provider yearly. If a participant did not have an eye care provider, the primary care professional would refer them. The study investigators contacted all the providers the participants could be referred to, asking them to complete the same assessment forms as done for those allocated to the intervention group. Participants in this group were also offered telemedicine screening after 48 months enrolment **Technical equipment used:** Digital non‐mydriatic fundus camera (model NM‐1000); Internal software for data transmission; Screen‐Vu stereoscope **Fidelity assessment:** Not reported	
Outcomes	**Main outcomes:** Percentage of participants receiving annual diabetic retinopathy screening examinations; percentage of eyes with worsening diabetic retinopathy; percentage of telemedicine participants who would require referral to an eye care professional for follow‐up care **Time points reported:** Baseline, follow‐up (12, 24, 36, and 48 months post‐baseline)	
Notes	**Funding:** National Eye Institute(NEI 3 K23 EY0155501‐01), the Centers for Disease Control and Prevention (CDCU48DP000024‐01 and 1U48DP002673‐01), and the Good Samaritan Foundation at Legacy Health **Ethical approval:** Institutional Review Boards of Legacy Health (Portland, OR),Oregon Health and Science University (Portland), and the Northwest Portland Area Indian Health Board (Portland). Trial registry: NCT01364129 **Conflicts of interest:** None known	
***Risk of bias***		
**Bias**	**Authors' judgement**	**Support for judgement**
Random sequence generation (selection bias)	Low risk	Quote: "We used a random number generator to randomly assign participants to the telemedicine group or the traditional surveillance group" (p.519)
Allocation concealment (selection bias)	Low risk	Quote: "We used a random number generator to randomly assign participants to the telemedicine group or the traditional surveillance group" (p.519)
Baseline outcome measurements similar (selection bias)	Unclear risk	Comment: Baseline measurements relating to service use outcome (i.e. previous attendance for screening) not reported. Baseline measurements relating to the clinical outcome (diabetic retinopathy) not reported
Baseline characteristics similar (selection bias)	Low risk	Comment: Baseline characteristics reported and similar between groups (Table 2)
Blinding of participants and personnel (performance bias) All outcomes	High risk	Comment: No information on blinding of central study personnel; not possible to blind primary care providers as they conducted the screening examinations; not possible to blind participants who received examinations in different settings
Blinding of objective outcome assessment (detection bias)	High risk	Comment: Due to the nature of the intervention it was not possible to blind participants or personnel
Protection against contamination	Unclear risk	Comment: No specific mention of measures to prevent contamination
Incomplete outcome data (attrition bias) All outcomes	Low risk	Comment: Low attrition rates for first 2 data points.
Selective reporting (reporting bias)	Unclear risk	Comment: Telemedicine was offered exclusively to the telemedicine group only to 2 years after recruitment. At 2 years standard‐care group also offered telemedicine. Yet follow‐up of exclusive telemedicine reported only to 18 months. Outcomes for worsening retinopathy reported for intervention and control groups combined
Other bias	High risk	Comment: Data collection methods differed for the 2 study groups. Whereas outcome data (screening attendance and clinical) in the telemedicine group were recorded by study staff (telemedical assessors, both report authors), the study relies upon eye‐care professionals in the community to report these for the standard care group. The researchers telephoned the community eye‐care professionals to introduce the project and request their participation in completing data collection forms for the study. It is uncertain how community eye‐care professional would know who was recruited in the study. Researchers also reviewed medical charts to search for eye examination data

**Table:** Orlandoni 2016

***Study characteristics***		
Methods	**Study design:** Randomised trial (parallel) **Unit of allocation:** Participant	
Participants	**Providers** **Number:** Not reported **Type:** Home‐visiting nursing staff consulting with a hospital physician **Other relevant characteristics:** Not reported **Participants** **Number:** Randomised: 188 (I: 100, C: 88), Analysed: unclear **Mean age (SD):** I. 86.5 (7.0), C: 84.4 (7.1) **Gender (% female):** I: 72%; C: 76% **Inclusion criteria:** Adults aged ≥ 65 years, attending the Department of Clinical Nutrition, treated with home enteral nutrition **Exclusion criteria:** Not reported **Other relevant characteristics:** Most had multiple morbidities **Location and study setting:** Italy, 1 hospital **Recruitment method:** Not reported **Duration:** Intervention lasted 12 months; study conducted between January and December 2013 **Withdrawals:** 38% and 33% of participants were lost to follow‐up (I and C, respectively), reasons not provided	
Interventions	**Intervention components:** Usual care plus video consultation ‐ during the monthly home visits, the home‐visiting staff called the hospital physician using the tablet. The latter would visually examine the participant for different clinical signs (e.g. hydration, oedema). Video calls lasted on average 2 minutes. If necessary, nutrition and pharmacological therapy would be adjusted **Comparison:** Usual care ‐ regular monthly home visits done by nurses to perform scheduled evaluations, which included an electrocardiogram, pulse oximetry, and blood glucose and pressure measurement. Data collected were logged online and reviewed by the hospital physician 2 to 3 days after the visit **Technical equipment used:** Tablet (Samsung Galaxy) **Fidelity assessment:** Video‐consultation followed a specific protocol; no further details provided	
Outcomes	**Main outcomes:** Frequency and type of complications; frequency and reason for outpatient visits and hospitalisations; modification of nutrition therapy; frequency and duration of video‐consultations (intervention group only) **Time points reported:** Baseline, follow‐up (12 months post‐baseline)	
Notes	**Funding:** Not reported **Ethical approval:** Hospital Ethics Committee **Conflicts of interest:** None known	
***Risk of bias***		
**Bias**	**Authors' judgement**	**Support for judgement**
Random sequence generation (selection bias)	Low risk	Quote: "Block randomisation (...) was carried out by the statistical service of [the hospital] using computer‐generated allocation" (p.763)
Allocation concealment (selection bias)	Low risk	Comment: Block randomisation was used
Baseline outcome measurements similar (selection bias)	Low risk	Comment: Baseline medical measurements that could impact the clinical outcome (main indicators of nutritional status, comorbidities, general health status) reported and similar between groups (Table 1)
Baseline characteristics similar (selection bias)	Low risk	Comment: Baseline characteristics provided and overall similar between groups; participants allocated to intervention were slightly older than those allocated to control
Blinding of participants and personnel (performance bias) All outcomes	High risk	Comment: Not possible to blind participants and personnel
Blinding of objective outcome assessment (detection bias)	High risk	Comment: Main outcome is overall complications. The home‐visit staff, who were not blinded, collected the outcome data
Protection against contamination	Unclear risk	Comment: Contamination between groups is possible as all home‐visiting staff and physicians had tablets which could be used with control participants
Incomplete outcome data (attrition bias) All outcomes	High risk	Comment: High rates of attrition, not explained
Selective reporting (reporting bias)	Low risk	Comment: All outcomes presented in the Methods are reported in the Results
Other bias	Low risk	Comment: No other apparent source of bias

**Table:** Pak 2007

***Study characteristics***		
Methods	**Study design:** Randomised trial (parallel) **Unit of allocation:** Participant	
Participants	**Providers** **Number:** Not reported **Type:** Primary care professional consulting with dermatologist **Other relevant characteristics:** Not reported **Participants** **Number:** Randomised: 698 (I: 351, C: 347), Analysed: 508: 236 in usual care and 272 in teledermatology **Mean age (SD):** I: 43.6 years; C: 46.8 years **Gender (% female):** I: 71%; C: 66% **Inclusion criteria:** Adults referred from Department of Defence primary care clinics **Exclusion criteria:** Urgent condition, multiple complaints **Other relevant characteristics:** Mainly white **Location and study setting:** USA, 4 primary care clinics (Department of Defence owned) **Recruitment method:** Eligible participants were invited to participate **Duration:** Single consultation with 4 months follow‐up; not reported when study was conducted **Withdrawals:** 33% of randomised participants did not complete follow‐up: 15% were withdrawn, mainly due to deployment or loss of privileges; 6% withdrew, mainly due to resolution of skin problem; 4% could not be contacted and were lost to follow‐up	
Interventions	**Intervention components:** A teledermatology appointment was scheduled, unclear how this was done. A dermatologist would then review the consultation and the images, and could either schedule a face‐to‐face appointment with the participant or send a diagnosis and management plan to the primary care professional **Comparison:** Usual care ‐ a dermatology appointment was scheduled at a clinic **Technical equipment used:** Digital camera (Coolpix 990, 3.3 megapixel); images were transferred using a web‐based secure server purposively developed **Fidelity assessment:** Data	
Outcomes	**Main outcomes:** Clinical improvement based on serial cutaneous examination; costs **Time points reported:** Baseline, post‐intervention (4 months post‐baseline)	
Notes	**Funding:** Telemedicine and Advanced Technology Research Center **Ethical approval:** Appropriate committees **Conflicts of interest:** "HSP is Chairman and Co‐founder of TeledermSolutions, Inc., a Web‐based teledermatology consultation service, is Co‐editor of Teledermatology: A user’s guide, published by Cambridge University Press, and is slated to receive royalties based on sales. JDW is Co‐editor of Teledermatology: A user’s guide, published by Cambridge University Press, and is slated to receive royalties based on sales."	
***Risk of bias***		
**Bias**	**Authors' judgement**	**Support for judgement**
Random sequence generation (selection bias)	Low risk	Comment: Authors report that block randomisation was used (p.27)
Allocation concealment (selection bias)	Low risk	Comment: Following informed consent the sealed envelope was opened to reveal the randomisation assignment
Baseline outcome measurements similar (selection bias)	Unclear risk	Comment: Not enough information provided
Baseline characteristics similar (selection bias)	High risk	Comment: Partciipants allocated to the control group were older than those allocated to intervention group (Table 1)
Blinding of participants and personnel (performance bias) All outcomes	High risk	Comment: Not possible to blind participants or personnel as care pathway was different
Blinding of objective outcome assessment (detection bias)	Low risk	Comment: Calculated using repayment rates
Blinding of subjective outcome assessment (detection bias)	Low risk	Quote: "A dermatologist blinded to randomisation assignment reviewed the images." (p.27)
Protection against contamination	Unclear risk	Comment: No information on strategies to prevent contamination or evidence suggesting contamination
Incomplete outcome data (attrition bias) All outcomes	High risk	Comment: High attrition rates
Selective reporting (reporting bias)	High risk	Comment: Outcomes reported in different papers
Other bias	Low risk	Comment: No other apparent risk of bias

**Table:** Piette 2017

***Study characteristics***		
Methods	**Study design:** Cluster‐randomised trial (parallel assignment) **Unit of allocation:** Cluster (8 primary care practices, 4 allocated to the intervention group and 4 allocated to the control group)	
Participants	**Providers** **Number:** 39 GPs, 3 dermatologists **Type:** GPs consulting with dermatologists **Other relevant characteristics:** Not reported **Participants** **Number:** Randomised: 109 (I:55, C:54), Analysed: 103 (I:53, C:50) **Mean age:** I: 44 years; C: 43.5 years **Gender (% female):** I: 70%; C: 50% **Inclusion criteria:** Adults with a skin condition for which the GP required a dermatologist's advice **Exclusion criteria:** Urgent medical care **Other relevant characteristics:** Not reported **Location and study setting:** France, 8 urban primary care practices **Recruitment method:** Participants identified by the GPs and invited to participate **Duration:** Single session; 90 days follow‐up **Withdrawals:** 5.5% of participants were excluded after being assessed as eligible (reasons provided)	
Interventions	**Intervention components:** GPs received training and a workbook on how to take photographs (p.2, top 2nd column). GPs took at least 3 photos of skin lesions and sent them with a standardised written message (date of symptoms, symptomatology, topography, description and extension of lesions, drug intake) through secure e‐mail to dermatologists. Dermatologists provided a diagnosis or possible differential diagnoses, and if necessary a management plan, which was implemented by the GP. The dermatologists could also book an appointment to see the participant in person **Comparison:** Usual care ‐ participants were given a standardised printed referral letter, which they could use to book an appointment with a dermatologist **Technical equipment used:** Photos were taken using either a mobile phone or digital camera (minimum 3 megapixels) **Fidelity assessment:** GPs received 2 hours training on how to take photos and were given a workbook explaining the detailed procedures to take photos compliant with the American Telemedicine Association recommendations	
Outcomes	**Main outcomes:** Days lapsed between the GP’s consultation and the dermatologist’s reply that allowed for the GP to begin treatment; participant's satisfaction; physicians' and participants' satisfaction; number of non‐usable photographs taken **Time points reported:** Post‐intervention (3 months post‐baseline)	
Notes	**Funding:** Pole de Santé Universitaire Gennevilliers Villeneuve la Garenne **Ethical approval:** Hospital Institutional Review Board. Trial registry: NCT02122432 **Conflicts of interest:** None known	
***Risk of bias***		
**Bias**	**Authors' judgement**	**Support for judgement**
Random sequence generation (selection bias)	Low risk	Comment: Computer‐generated random list (p.2)
Allocation concealment (selection bias)	Low risk	Comment: Investigator generated the list at the start of the study for all primary practices; investigator did not have contact with physicians or participants (p.2)
Baseline outcome measurements similar (selection bias)	Unclear risk	Comment: Not enough information provided
Baseline characteristics similar (selection bias)	High risk	Comment: Baseline characteristics provided, groups different for sex distribution and dermatologist final diagnosis (Table 1)
Blinding of participants and personnel (performance bias) All outcomes	High risk	Comment: Participants, GPs, and study personnel were not blinded to group allocation (p.2)
Blinding of objective outcome assessment (detection bias)	High risk	Comment: Days between consultations reported by the participant, who was not blinded to group allocation
Blinding of subjective outcome assessment (detection bias)	High risk	Comment: Reported by GPs and participants who were not blinded to group allocation, and dermatologists (intervention group only)
Protection against contamination	Low risk	Comment: Allocation by GP practices
Incomplete outcome data (attrition bias) All outcomes	Low risk	Comment: Low attrition rates (Figure 1)
Selective reporting (reporting bias)	Low risk	Comment: All outcomes specified in the protocol were reported in the published article
Other bias	Low risk	Comment: No other apparent risk of bias

**Table:** Riordan 2015

***Study characteristics***		
Methods	**Study design:** Randomised trial (cross‐over) **Unit of allocation:** Participant	
Participants	**Providers** **Number:** 8 emergency department (ED) residents **Type:** ED residents consulting with consultants **Other relevant characteristics:** Not reported **Participants** **Number:** Not reported **Mean age (SD):** Not reported **Gender (% female):** Not reported **Inclusion criteria:** Adults attending the ED **Exclusion criteria:** Not reported **Other relevant characteristics:** Not reported **Location and study setting:** USA, 1 ED **Recruitment method:** Not reported **Duration:** Single consultation with 1 month follow‐up; not reported when study was conducted **Withdrawals:** Not reported	
Interventions	**Intervention components:** Electronic consultation application, used by the ED resident to communicate with consultants **Comparison:** Usual care **Technical equipment used:** Tablets (iPad) **Fidelity assessment:** Not reported	
Outcomes	**Main outcomes:** Conciseness; pertinence of information presented; flow; effectiveness of communication skills; overall quality of physician to physician consultations **Time points reported:** Post‐intervention (1 month post‐baseline)	
Notes	**Funding:** Not reported **Ethical approval:** Not reported **Conflicts of interest:** Not reported	
***Risk of bias***		
**Bias**	**Authors' judgement**	**Support for judgement**
Random sequence generation (selection bias)	Unclear risk	Comment: Not enough information provided
Allocation concealment (selection bias)	Unclear risk	Comment: Not enough information provided
Baseline outcome measurements similar (selection bias)	Unclear risk	Comment: Not enough information provided
Baseline characteristics similar (selection bias)	Unclear risk	Comment: Not enough information provided
Blinding of participants and personnel (performance bias) All outcomes	Unclear risk	Comment: Not enough information provided
Blinding of objective outcome assessment (detection bias)	Unclear risk	Comment: Not enough information provided
Blinding of subjective outcome assessment (detection bias)	Unclear risk	Comment: Not enough information provided
Protection against contamination	Unclear risk	Comment: Not enough information provided
Incomplete outcome data (attrition bias) All outcomes	Unclear risk	Comment: Not enough information provided
Selective reporting (reporting bias)	Unclear risk	Comment: Not enough information provided
Other bias	Unclear risk	Comment: Not enough information provided

**Table:** Sutherland 2009

***Study characteristics***		
Methods	**Study design:** Randomised trial (parallel) **Unit of allocation:** Participant	
Participants	**Providers** **Number:** 1 primary care physician, 6 radiologists **Type:** Primary care physician consulting with radiologists **Other relevant characteristics:** Primary care physician received sonographic training at 3 US medical centres **Participants** **Number:** Randomised: 105 (I: 53, C: 52), Analysed: same number **Mean age (SD):** I: 27 years; C: 29 years **Gender (% female):** I: 90; C: 94% **Inclusion criteria:** Participants aged ≥ 13 years attending a primary care clinic, with symptoms requiring a trans‐abdominal or trans‐vaginal ultrasound **Exclusion criteria:** Not reported **Other relevant characteristics:** Low‐income setting **Location and study setting:** Dominican Republic, 1 rural clinic **Recruitment method:** All eligible patients were invited to participate **Duration:** Intervention was 1 consultation; not reported when study was conducted **Withdrawals:** No withdrawals	
Interventions	**Intervention components:** Primary care professional performed scans according to current practice guidelines, which were then emailed to US‐based radiologists along with forms with any relevant clinical information. The on‐site investigator received sonographic training over a 2‐month period, as well as practice guidelines for trans‐abdominal ultrasound scanning. The radiologists interpreted the scans and returned the forms, along with an assessment of the scan's quality. Participants were instructed to return to the primary care clinic within 48 hours **Comparison:** Usual care ‐ received regular ultrasound referral and were instructed to return the diagnostic report in hand as soon as possible **Technical equipment used:** Portable ultrasound scanner (SonoSite Titan with 5.2 MHz curvilinear transducer), images sent by email as attachment **Fidelity assessment:** Not reported	
Outcomes	**Main outcomes:** Time to final diagnosis; time to follow‐up appointments; number of successful follow‐ups; number of delivered reports **Time points reported:** Post‐intervention	
Notes	**Funding:** Global Health Leadership Fellowship, sponsored by the Edward Via Virginia College of Osteopathic Medicine **Ethical approval:** Appropriate ethics committee **Conflicts of interest:** Not reported	
***Risk of bias***		
**Bias**	**Authors' judgement**	**Support for judgement**
Random sequence generation (selection bias)	High risk	Comment: Coin tossing (p.192)
Allocation concealment (selection bias)	High risk	Comment: Coin tossing is at high risk of allocation being predictable.
Baseline outcome measurements similar (selection bias)	Unclear risk	Comment: Not enough information provided
Baseline characteristics similar (selection bias)	Unclear risk	Comment: Not enough information provided
Blinding of participants and personnel (performance bias) All outcomes	High risk	Comment: Only 1 primary care physician, not possible to blind; participants were aware of group allocation as it implied different actions
Blinding of objective outcome assessment (detection bias)	Low risk	Comment: The radiologists were blinded from one another’s interpretations (p.194)
Protection against contamination	Low risk	Comment: Not possible for contamination to occur
Incomplete outcome data (attrition bias) All outcomes	Low risk	Comment: No attrition
Selective reporting (reporting bias)	Low risk	Comment: All outcomes mentioned in the Methods are reported in the Results section
Other bias	Low risk	Comment: No other apparent risk of bias

**Table:** Taylor‐Gjevre 2018

***Study characteristics***		
Methods	**Study design:** Randomised trial (parallel) **Unit of allocation:** Participant	
Participants	**Providers** **Number:** **Type:** 3 rural‐based physical therapists consulting with 3 urban‐based rheumatologists **Other relevant characteristics:** Not reported **Participants** **Number:** Randomised: 85 (I: 54, C: 31), Analysed: 54 (I: 31, C: 23) **Mean age (SD):** I: 58.4 years (10.7); C: 53.1 years (12.2) **Gender (% female):** I: 80%; C: 81% **Inclusion criteria:** Adults with a clinical diagnosis of rheumatoid arthritis, living more than 100 km away from the urban centres **Exclusion criteria:** Not reported **Other relevant characteristics:** Mean duration of rheumatoid arthritis was 1.9 years **Location and study setting:** Canada, 1 urban clinic, 5 rural clinics **Recruitment method:** Identified through the clinic databases **Duration:** Intervention was 1 consultation every 3 months, follow‐up lasted 9 months; not reported when study was conducted **Withdrawals:** 43% (I) and 26% (C) of participants did not complete the study; main reason provided by participants allocated to the intervention group was a preference for travelling into town for their appointment	
Interventions	**Intervention components:** Video‐consultations between physical therapist and rheumatologist; the participants were present for part of the consultation, during which they were examined by the rheumatologist. Physical therapists and rheumatologists received an orientation and education session about rheumatoid arthritis and the study protocol and methods **Comparison:** Usual care ‐ in‐person rheumatology clinics **Technical equipment used:** Laptops with video‐conferencing software (VidyoDesktop software); detachable external web camera with remote pan, tilt and zoom functions **Fidelity assessment:** All healthcare professionals attended an education session about the study protocol	
Outcomes	**Main outcomes:** Disease activity metrics; health assessment; participant satisfaction **Time points reported:** Baseline, post‐intervention (9 months post‐baseline)	
Notes	**Funding:** Canadian Initiative for Outcomes in Rheumatology cAre (CIORA) **Ethical approval:** University of Saskatchewan Biomedical Research Ethics Board; trial registry NCT02371915 **Conflicts of interest:** Not reported	
***Risk of bias***		
**Bias**	**Authors' judgement**	**Support for judgement**
Random sequence generation (selection bias)	Low risk	Quote: "[S]tratified block randomisation algorithm" (p.2)
Allocation concealment (selection bias)	Low risk	Quote: "Clinicians were not involved in or aware of the outcome of the randomisation allocation, which was overseen by the research coordinator" (p.2)
Baseline outcome measurements similar (selection bias)	Low risk	Comment: Baseline outcome measurements reported and similar between groups (Fig. 2)
Baseline characteristics similar (selection bias)	Low risk	
Blinding of participants and personnel (performance bias) All outcomes	High risk	Comment: Due to the nature of the intervention participants and personnel could not have been blinded and no attempts at blinding were described
Blinding of objective outcome assessment (detection bias)	High risk	Comment: Physical examination data were collected by on‐site physical therapists and by urban rheumatologists, due to the nature of the intervention they could not have been blinded
Blinding of subjective outcome assessment (detection bias)	High risk	Comment: Self‐reported outcomes were quality of life and satisfaction with care, and participants were not blinded
Protection against contamination	Unclear risk	Comment: Many of the dropouts were reportedly due to preference for standard treatment
Incomplete outcome data (attrition bias) All outcomes	High risk	Comment: High attrition rates
Selective reporting (reporting bias)	High risk	Comment: Reports all outcomes mentioned in Methods section of paper, but not all outcomes stated in online trial record (NCT02371915), e.g. change in healthcare use
Other bias	Low risk	Comment: No other apparent risk of bias

**Table:** Van Gelder 2017

***Study characteristics***		
Methods	**Study design:** Cluster‐randomised trial (parallel) **Unit of allocation:** Cluster (47 primary care practices, 23 allocated to the intervention group and 24 to the control group)	
Participants	**Providers** **Number:** 128 GPs (number of nephrologists not provided) **Type:** GPs consulting with nephrologists **Other relevant characteristics:** Not reported **Participants** **Number:** I: 1277; C: 1727 **Mean age (SD):** I: 68.0 years (13.6); C: 66.4 years (13.2) **Gender (% female):** I: 67%; C: 65% **Inclusion criteria:** Adults with a clinical diagnosis of chronic kidney disease who qualified for consultation or referral to nephrology specialist care **Exclusion criteria:** Receiving secondary renal care **Other relevant characteristics:** Most had at least 1 comorbid chronic condition **Location and study setting:** The Netherlands, 47 primary care practices across the country **Recruitment method:** GPs were invited to participate while attending a CKD management course; eligible patients were identified through EMR **Duration:** Intervention was implemented between March 2011 and June 2012; follow‐up duration unclear **Withdrawals:** Approximately 3% of eligible patients did not start the trial (reasons provided); 7.7% of participants did not complete follow‐up (deceased: n = 181; moved: n = 50; unknown: n = 1)	
Interventions	**Intervention components:** Telenephrology was added to the EMR as an add‐on application, which was activated by the GP for each specific participant. The nephrologist was then notified about the consultation by e‐mail or text message and advised the GP about further treatment required, including referrals if needed **Comparison:** Usual care ‐ conventional consultation methods **Technical equipment used:** Encrypted EMR, accessed with a direct single sign‐on **Fidelity assessment:** Not reported	
Outcomes	**Main outcomes:** Difference in referral rate between intervention and control groups **Other outcomes:** Difference in consultation rates by telephone or telenephrology; adherence to the advised monitoring criteria; GP's compliance with coding renal impairment as a separate entity; achievement of blood pressure targets; main related medical costs; incidence of CKD; GPs experience with using telenephrology **Time points reported:** Unclear	
Notes	**Funding:** Dutch Kidney Foundation and Amgen **Ethical approval:** Not required according to the accredited Medical Research Ethics Committee Arnhem/Nijmegen. Clinicians and participants were informed electronic medical data were being used for research purposes and could opt‐out. Netherlands Trial Registration 2242 **Conflicts of interest:** "The Department of Primary and Community Care received a non‐conditional grant from Amgen. Jack Wetzels received research grants from Amgen, Genzyme and Pfizer for the Masterplan study. All other authors have no conflicting interests"	
***Risk of bias***		
**Bias**	**Authors' judgement**	**Support for judgement**
Random sequence generation (selection bias)	Low risk	Comment: Stratified block randomisation (p.432)
Allocation concealment (selection bias)	Low risk	Comment: Independent statistician performed randomisation by institution at the start of the study (p.432)
Baseline outcome measurements similar (selection bias)	Unclear risk	Comment: Not enough information provided to make a decision
Baseline characteristics similar (selection bias)	Low risk	Comment: Baseline characteristics provided and similar between groups (table 1)
Blinding of participants and personnel (performance bias) All outcomes	High risk	Comment: Due to the nature of the intervention participants and personnel could not have been blinded and no attempts at blinding were described
Blinding of objective outcome assessment (detection bias)	High risk	Comment: All referrals were reported by both the GPs and the nephrologists in an online survey system
Blinding of subjective outcome assessment (detection bias)	High risk	Comment: GPs in the intervention group answered a survey about their experience with the intervention (p.432)
Protection against contamination	High risk	Comment: GPs allocated to the CG participated in a training course about CKD (p.435)
Incomplete outcome data (attrition bias) All outcomes	Low risk	Comment: Low attrition rates
Selective reporting (reporting bias)	Low risk	Comment: All outcomes specified in the protocol were reported in the published article
Other bias	Low risk	Comment: No other apparent risk of bias

**Table:** Whited 2002

***Study characteristics***		
Methods	**Study design:** Randomised trial (parallel) **Unit of allocation:** Participant	
Participants	**Providers** **Number:** 60 GPs, 8 dermatologists **Type:** GPs consulting with dermatologists **Other relevant characteristics:** Dermatologists were mainly third‐year residents **Participants** **Number:** Randomised: 274 (I: 134, C: 140), Analysed: ITT analysis **Mean age (SD):** I: 60.9 years (7.8); C: 66.9 years (8.5) **Gender (% female):** 5% **Inclusion criteria:** Adults referred to the Dermatology service from primary care clinics **Exclusion criteria:** Urgent conditions that required immediate attention **Other relevant characteristics:** Mainly white **Location and study setting:** USA, 4 clinics at a Veteran Affairs Medical Centre **Recruitment method:** Not reported **Duration:** 1 consultation/referral; not reported when study was conducted **Withdrawals:** Not reported	
Interventions	**Intervention components:** GPs submitted digital images of skin lesions with a standardised medical history and any additional relevant information. The consultant dermatologist reviewed all the data and replied either by scheduling a clinic‐based appointment or sending a diagnosis and management plan to the GP, without further need for a clinic‐based appointment **Comparison:** Usual care ‐ GPs referred participants to the dermatology service as needed **Technical equipment used:** Fujix DS‐515 digital camera **Fidelity assessment:** As a quality‐control measure, images were assessed on a laptop computer while acquiring them	
Outcomes	**Main outcomes:** Time to intervention; costs; participant and healthcare professional satisfaction **Time points reported:** Baseline, resolution of the problem (variable)	
Notes	**Funding:** VA Health Services Research and Development Service and VA Health Services Research and Development Service Research Career Development Award **Ethical approval:** Research and Development Committee and the Human Studies Subcommittee of the Department of Veterans Affairs Medical Center, Durham, North Carolina **Conflicts of interest:** Not reported	
***Risk of bias***		
**Bias**	**Authors' judgement**	**Support for judgement**
Random sequence generation (selection bias)	Unclear risk	Comment: Not enough information provided
Allocation concealment (selection bias)	Low risk	Quote: "Referring primary care clinicians contacted the research assistants during the course of clinic visits when a dermatology consult was considered appropriate. Research assistants were blinded to the study arm in which prospective patients were randomised." (p.314)
Baseline outcome measurements similar (selection bias)	Low risk	Comment: Lesion characteristics were similar between groups (Table 2)
Baseline characteristics similar (selection bias)	Unclear risk	Comment: Baseline characteristics reported and similar between groups (Table 2)
Blinding of participants and personnel (performance bias) All outcomes	High risk	Comment: Not possible to blind participants and personnel
Blinding of objective outcome assessment (detection bias)	Low risk	Quote: "Research assistants were blinded to the study arm in which prospective patients were randomised." (p.314)
Blinding of subjective outcome assessment (detection bias)	High risk	Comment: Self‐reported satisfaction
Protection against contamination	Unclear risk	Comment: Contamination unlikely to be a risk at participant level. Risks of or strategies to prevent contamination at the level of responding dermatologists unclear
Incomplete outcome data (attrition bias) All outcomes	Low risk	Comment: Data analysed for all participants
Selective reporting (reporting bias)	High risk	Comment: Different outcomes reported in different publications
Other bias	Low risk	Comment: No other apparent risk of bias

**Table:** Whited 2013

***Study characteristics***		
Methods	**Study design:** Randomised trial (parallel) **Unit of allocation:** Participant	
Participants	**Providers** **Number:** Not reported **Type:** GPs consulting with dermatologists **Other relevant characteristics:** Not reported **Participants** **Number:** Randomised: 392 (I: 196, C: 196), Analysed: 261 (I: 136; C: 125) **Mean age (SD):** I: 62.9 years (13.9); C: 61.7 years (14.9) **Gender (% female):** 2% (Veteran Affairs clinics) **Inclusion criteria:** Adults referred to the Dermatology service from primary care clinics **Exclusion criteria:** More than 1 skin condition, required full‐body examination, could not read or speak English, low health literacy **Other relevant characteristics:** Mainly white **Location and study setting:** USA, 2 outpatient community‐based Veteran Affairs clinics **Recruitment method:** Eligible participants were identified whenever the GP generated a request for a consultation with the dermatology department **Duration:** 1 consultation/referral (unless participant required further treatment), 9 months follow‐up; study conducted between November 2008 and March 2011 **Withdrawals:** 33% of participants randomised did not complete follow‐up (reasons provided, similar numbers for I and C)	
Interventions	**Intervention components:** Alongside the request for a referral, GPs submitted digital images of skin lesions with a standardised medical history and any additional relevant information. The consultant dermatologist reviewed all the data and replied either by scheduling a clinic‐based appointment or sending a diagnosis and management plan to the GP, without further need for a clinic‐based appointment **Comparison:** Usual care ‐ GPs referred participants to the dermatology service as needed using the electronic medical record **Technical equipment used:** 8‐megapixel digital camera with an integrated flash; if required, digital ring flash for short focal length or macro images **Fidelity assessment:** Imaging protocol	
Outcomes	**Main outcomes:** Quality of life; health status; comorbidity assessment; cost; satisfaction with care **Time points reported:** Baseline, follow‐up (3 and 9 months post‐baseline assessment)	
Notes	**Funding:** US Department of Veterans Affairs Health Services Research and Development Service; National Institutes of Health **Ethical approval:** Approved by institutional review boards. Trial registry: NCT00488293 **Conflicts of interest:** "Drs Whited and Edison are coeditors of the book Teledermatology: A User’s Guide published by Cambridge University Press and receive royalties based on sales. Dr Chren is a consultant to Genetech Inc (on patient‐reported outcomes)."	
***Risk of bias***		
**Bias**	**Authors' judgement**	**Support for judgement**
Random sequence generation (selection bias)	Low risk	Comment: Simple randomisation scheme stratified by site (p.586)
Allocation concealment (selection bias)	Low risk	Comment: Off‐site statistical co‐ordinating centre (p.586)
Baseline outcome measurements similar (selection bias)	Low risk	Comment: Baseline outcome measurements provided and similar between groups (table 1)
Baseline characteristics similar (selection bias)	Low risk	Comment: Baseline outcome characteristics provided and similar between groups (table 1)
Blinding of participants and personnel (performance bias) All outcomes	High risk	Comment: Not possible due to the nature of the intervention
Blinding of objective outcome assessment (detection bias)	High risk	Comment: Costs partially calculated based on site reports, which are variable; clinical staff not blinded to group allocation
Blinding of subjective outcome assessment (detection bias)	High risk	Comment: Participants, clinical staff and research personnel not blinded to group allocation
Protection against contamination	Unclear risk	Comment: Contamination unlikely to be a risk at participant level. Risks of or strategies to prevent contamination at the level of responding dermatologists unclear
Incomplete outcome data (attrition bias) All outcomes	High risk	Comment: High attrition rates (67% participants randomised were included in primary analysis)
Selective reporting (reporting bias)	High risk	Comment: Outcomes differ between protocol and publications
Other bias	Low risk	Comment: No other apparent risk of bias

Empty cells in the "Risk of bias" tables refer to instances where the specific risk of bias criterion did not apply, e.g. the type of outcome was not collected by the study. ART: Antiretroviral Therapy; C: Control; CKD: Chronic kidney disease; EMR: Electronic medical record; GPs: General practitioners; HCP: Healthcare provider; HEW: Health extension workers; I: Intervention; PCP: Primary care provider; PHWs: Peer health workers; VA: Veteran Affairs

### Characteristics of excluded studies [ordered by study ID]

**Table:**

Study	Reason for exclusion
Ateudjieu 2014	Compares in‐person supervision with automated text messages
Atnafu 2017	Mobile technologies used for emergency referrals, not seeking guidance or providing care
Batista 2016	Not mobile
Bettinelli 2015	Feasibility study
Burns 2016	Not mobile (desktop video‐conferencing system)
Buvik 2016	Not mobile (desktop personal computers)
Chiaravalloti 2017	Pilot study
Conlin 2006	Not an intervention study (diagnostic accuracy)
Da Silva 2018	Not mobile
Ferrándiz 2017	Compares 2 Internet‐based interventions (clinical images vs. dermoscopic images)
Golberstein 2017	Not mobile (desktop personal computers)
Gong 2018	Multifaceted study with several components
Haridy 2017	Pilot study
Loane 2001	Not mobile (desktop video‐conferencing telephone)
NCT02710799	Not mobile
Nwando Olayiwola 2016	Not mobile (desktop personal computers, as well as laptops)
Oakley 2000	Not mobile (desktop personal computers)
Owen 2019	Feasibility study
Phillips 2019	Mainly educational
Pryzbylo 2014	Compares 2 devices (smartphone and pager) for routine communication
Romero 2009	Diagnostic accuracy study
Wesarg 2010	Compares 2 methods for fitting a Cochlear device, each participant has the device fitted remotely or face‐to‐face (not randomised)

### Characteristics of ongoing studies [ordered by study ID]

**Table:** ACTRN12617000389303

Study name	Establishing the role of teleconsulting in the care of chronic conditions in rural areas of the Southern District Health Board (SDHB): A randomised controlled trial (RCT) in patients with Inflammatory Bowel Disease
Methods	Randomised trial, parallel assignment, open‐label
Participants	Adults aged ≥ 18 years diagnosed with irritable bowel syndrome living in rural settings
Interventions	**Intervention:** remote consultation through teleconference with nurse facilitation **Comparison:** usual care
Outcomes	**Main outcomes:** disease control; disease‐specific quality of life **Other outcomes:** cost effectiveness; acceptability
Starting date	April 2017 (expected completion date June 2020)
Contact information	Ms Christine Ho (Christine.Ho@otago.ac.nz)
Notes

**Table:** ACTRN12618001007224

Study name	A prospective randomised controlled study of telehealth specialist palliative care consultations in rural and metropolitan settings and the impact on patient and carer clinical outcomes and quality‐of‐life
Methods	Randomised trial, parallel assignment, open‐label
Participants	Adults aged ≥ 18 years receiving community, inpatient or outpatient palliative care
Interventions	**Intervention:** in‐home consultation through teleconference with nurse facilitation **Comparison:** usual care
Outcomes	**Main outcomes:** clinical symptoms; quality of life; performance status **Other outcomes:** emergency department attendances; time to set up teleconference equipment; user experience; home visit duration; other participant‐reported symptoms
Starting date	June 2018 (expected completion date October 2020)
Contact information	A/Prof Peter Poon (peter.poon@monashhealth.org)
Notes

**Table:** Done 2018

Study name	Teledermatology mobile apps: implementation and impact on veterans' access to dermatology
Methods	Randomised trial, cross‐over assignment, open‐label
Participants	All people receiving dermatology care at eligible clinics
Interventions	**Intervention:** Tablet loaded with app, which allows to capture and immediately upload images into the electronic records system, where it can be reviewed by referring providers and imagers **Comparison:** Usual care (for 3‐month blocks, until they also start using the app)
Outcomes	**Main outcomes:** consult completion time; appointment completion time; number of teledermatology appointments; fraction of appointments using teledermatology; travel distance **Other outcomes:** none specified
Starting date	March 2019 (estimated completion date September 2020)
Contact information	Dennis Oh (dennis.oh@va.gov)
Notes	Published protocol

**Table:** Gervès‐Pinquié 2017

Study name	CAPRI
Methods	Randomised trial, parallel assignment, open‐label
Participants	People with metastatic cancer or haematological malignancy being treated with oral therapy, aged ≥ 18 years
Interventions	**Intervention:** Participants will be given access to nurse navigators and a web portal, which will also be used for nurses to communicate with other healthcare professionals **Comparison**: Usual care
Outcomes	**Main outcomes:** relative dose intensity **Other outcomes:** compliance; toxicity
Starting date	October 2016 (estimated completion date October 2020)
Contact information	Marie Ferrua (marie.ferrua@gustaveroussy.fr)
Notes	Registered trial (NCT02828462)

**Table:** Jeandidier 2018

Study name	Evaluation of the DIABEO system in poorly controlled DM1 or DM2 patients treated with a basal‐bolus insulin regimen
Methods	Randomised trial, parallel assignment, open‐label
Participants	People aged ≥ 18 years with Type 1 or Type 2 diabetes
Interventions	**Intervention:** Participants are provided with an electronic diary system for monitoring glycaemic levels; results are uploaded to an online portal that can be accessed by HCP; clinical information can be exchanged between different HCPs **Comparison**: Usual care
Outcomes	**Main outcomes:** change in HbA1c **Other outcomes:** HbA1c levels; percent of responder participants; severe hypoglycaemia
Starting date	February 2013 (estimated completion date July 2018)
Contact information	Sylvia Franc
Notes	No published results found, contact authors emailed twice for further information, no reply Registered trial (NCT02287532)

**Table:** Koch 2018

Study name	TeleDerm study
Methods	Cluster‐randomised trial, parallel assignment, open‐label
Participants	Adults with a dermatologic problem and insured by a specific health insurance company
Interventions	**Intervention:** When faced with a dermatologic case, the GP can trigger a teleconsultation process with a dermatologist, based on high‐resolution pictures and clinical history **Comparison**: Usual care
Outcomes	**Main outcomes:** Number of physical referrals to dermatologists **Other outcomes:** Referral time; process quality; health‐related quality of life; costs
Starting date	July 2018 (expected completion date June 2019)
Contact information	Roland Koch (roland.koch@med.uni‐tuebingen.de)
Notes	Registered trial (DRKS00012944)

**Table:** Källander 2015

Study name	inSCALE
Methods	Randomised trial (cluster parallel)
Participants	Community health workers (CHWs) working in districts with Integrated Community Case Management (Uganda and Mozambique)
Interventions	CHWs are equipped with smartphones that can be used to facilitate decision‐making, submit data, receive personal performance feedback and communicate with their supervisor
Outcomes	**Main outcome:** appropriate treatment of malaria, pneumonia and diarrhoea in children under 5 years of age at 12 months **Other outcomes:** CHWs with medicine stock‐out < 1 week each quarter; CHW retention
Starting date	April 2013
Contact information	Karen Kalländer
Notes	Registered trial (NCT01972321)

**Table:** Nakayama 2016

Study name	Screening of cardiovascular, cerebrovascular, and renal disease for residents in rural areas using a medical IT network
Methods	Randomised trial, parallel assignment, open‐label
Participants	Adults aged ≥ 65 years living in rural areas, with low‐to‐moderate risk of cardiovascular disease
Interventions	**Intervention:** Using clinical data from a medical information network, specialists in cardiology, nephrology and cerebrovascular disease assess patient data and make treatment recommendations to GPs **Comparison**: Usual care, participants are treated in‐person by physician
Outcomes	**Main outcomes:** Incidence of cardiovascular, cerebrovascular, or renal disease **Other outcomes:** Not reported
Starting date	May 2015 (no information about study completion)
Contact information	Masaharu Nakayama (nakayama@cardio.med.tohoku.ac.jp)
Notes	No published results found, contact authors emailed twice for further information, no reply Registered trial (UMIN000018552)

**Table:** NCT02821143

Study name	The impact of Telemedicine to support palliative care resident in nursing home (TELESM)
Methods	Randomised trial, parallel assignment, open‐label
Participants	Nursing home residents aged ≥ 65 years, with palliative care needs
Interventions	**Intervention:** Telemedicine consultation ‐ multi‐professional consultation with healthcare professionals (participant and their families can also participate if desired) **Comparison:** Usual care
Outcomes	**Main outcomes:** hospitalisation rates **Other outcomes:** emergency hospitalisation rates; proportion of hospitalised participants; quality of life; caregiver satisfaction; costs
Starting date	January 2018
Contact information	Sandrine Sourdet (sourdet.s@chu‐toulouse.fr)
Notes

**Table:** NCT02986256

Study name	Evaluation of the management of diabetic foot ulcers by telemedicine on the number of hospital days in diabetic patients (TELEPIED)
Methods	Randomised trial, parallel assignment, open‐label
Participants	Patiients with diabetes and foot ulcer aged ≥ 18 years
Interventions	**Intervention:** During home visits the community nurse will photograph foot ulcers, which will be sent to the specialist nurse for assessment and follow‐up **Comparison:** Usual care
Outcomes	**Main outcomes:** number of hospitalisation days due to diabetic foot ulcers **Other outcomes:** total direct care costs; average duration of hospitalisation due to diabetic foot ulcers; ulcer recidivism rate; frequency of ulceration; duration of ulceration; healing rate; amputation rate; participant satisfaction score
Starting date	January 2017 (estimated completion date January 2021)
Contact information	Sylvia Franc (sylvia.franc@free.fr)
Notes

**Table:** NCT03137511

Study name	OASE Melanome
Methods	Cluster‐randomised trial, open‐label
Participants	Adults aged ≥ 18 years consulting a GP for a suspicious cutaneous lesion who require a referral to a dermatologist
Interventions	**Intervention:** The GP sends the dermatologist 2 photos of skin lesions, along with relevant clinical information, after which the dermatologist assesses the photos and follows up with the participant as required **Comparison:** Usual care
Outcomes	**Main outcomes:** Time limit between consultation with GP and consultation with dermatologist **Other outcomes:** Proportion of participants who did have a consultation with a dermatologist 12 months after consulting with GP
Starting date	May 2017 (estimated completion date May 2018)
Contact information	Jean‐Michel Nguyen (jeanmichel.nguyen@chu‐nantes.fr)
Notes	No published results found, contact authors emailed twice for further information, no reply

**Table:** NCT03559712

Study name	Effectiveness of collaborative tele‐mental health services for ADHD in primary care: a randomised trial in Dubai (ECTSAP‐ Dubai Trial)
Methods	Randomised trial, parallel assignment, open‐label
Participants	Children aged 6 to 12 years diagnosed with attention deficit hyperactivity disorder (ADHD)
Interventions	**Intervention:** remote consultation through teleconference with specialist supervision **Comparison:** usual care
Outcomes	Change in clinical symptoms
Starting date	June 2018 (expected completion date December 2018, personal communication with principal investigator 22 October 2019 confirmed it is ongoing)
Contact information	Ammar AlBanna (aalbanna@ajch.ae)
Notes

**Table:** NCT03662256

Study name	Addressing early childhood hearing loss in rural Alaska: a community randomised trial
Methods	Randomised trial, parallel assignment, single masking (outcomes assessor)
Participants	Children aged 2 to 6 years, attending eligible schools
Interventions	Little information provided; intervention described as telemedicine referral and mHealth screening tool
Outcomes	**Main outcomes:** time to diagnosis **Other outcomes:** sensitivity and specificity of screening protocols; prevalence of hearing loss
Starting date	September 2018 (estimated completion date February 2020)
Contact information	Samantha Robler (skleindienst@nshcorp.org)
Notes

**Table:** Stevanovic 2017

Study name	Telemedical support for prehospital Emergency Medical Service (TEMS)
Methods	Randomised trial, parallel assignment, open‐label
Participants	All emergency calls that are assessed as non‐life‐threatening, which do not require an obligatory emergency medical service physician on scene and which do not solely require an ambulance staffed by paramedics
Interventions	**Intervention:** Tele‐EMS physician ‐ participants are treated by the paramedics, who will be supported by the tele‐EMS physicians based at a teleconsultation centre **Comparison**: Usual care, participants are treated by physician on scene
Outcomes	**Main outcomes:** adverse events **Other outcomes:** adherence to guidelines; quality of medical history; completeness and correctness of data; tracer diagnoses; mortality; intensive care unit length of stay; hospital length of stay; other outcomes
Starting date	July 2017 (estimated study completion date December 2019)
Contact information	Ana Stevanovic (astevanovic@ukaachen.de)
Notes	Published protocol

**Table:** Xu 2017

Study name	A coordinated PCP‐cardiologist telemedicine model (PCTM) in China's community hypertension care
Methods	Randomised trial, parallel assignment, open‐label
Participants	Adults aged ≥ 21 years, with a clinical diagnosis of hypertension with uncontrolled blood pressure in the past 3 months, currently taking or about to take anti‐hypertensive medications
Interventions	**Intervention:** Participants are given a blood pressure monitoring system for self‐management, which feeds data back to the primary care and cardiology team. Primary care providers and cardiologists use a web‐based system to communicate and manage care **Comparison:** Usual care ‐ based on national guidelines for hypertension management
Outcomes	**Main outcomes:** Changes in mean systolic blood pressure **Other outcomes:** Changes in mean diastolic blood pressure; hypertension control rate; medication adherence
Starting date	September 2016 (estimated completion date August 2018)
Contact information	Lei Xu (waqyl@126.com)
Notes	No published results found, contact authors emailed twice for further information, no reply. Trial registry NCT02919033

DM1 or DM2: Type 1 or Type 2 Diabetes Mellitus; EMS: Emergency medical service; GP: General practitioner; HCP: Healthcare professionals

## Differences between protocol and review

We stated in the protocol that there would be one 'Summary of findings' table per comparison, and that we would group the trials by health condition. We did not do this due to the low number of included trials and the large number of different health conditions. Following discussion among the authors we agreed that stratifying the included studies by setting (community, primary, secondary care) would provide relatively homogenous groups of studies and that reporting findings by setting would improve the usability of the evidence.

We changed the title from "Mobile‐based technologies to support healthcare provider to healthcare provider communication and management of care" to "Mobile technologies to support healthcare provider to healthcare provider communication and management of care".

For the outcome 'Healthcare provider and participant acceptability of and satisfaction with the intervention', the protocol stated that both objective and subjective measures would be included, the former being the number lost to follow‐up not explained by other reasons. We did not measure acceptability or satisfaction using loss to follow‐up data, due to insufficient information.

We split the outcome 'Resource use' into two outcomes ('Healthcare use' and 'Cost').

For the 'Summary of findings' tables, we included participant acceptability and satisfaction alongside healthcare provider acceptability and satisfaction. The former was already a prespecified outcome in the protocol, but not for the 'Summary of findings' table.

One of the authors left the team (Nicola Maayan); we added new authors (Ana Rita Maria, Ignacio Ricci‐Cabello, Gemma Villanueva).
